# Rho-Associated Kinases and Non-muscle Myosin IIs Inhibit the Differentiation of Human iPSCs to Pancreatic Endoderm

**DOI:** 10.1016/j.stemcr.2017.07.005

**Published:** 2017-08-08

**Authors:** Taro Toyoda, Azuma Kimura, Hiromi Tanaka, Tomonaga Ameku, Atsushi Mima, Yurie Hirose, Masahiro Nakamura, Akira Watanabe, Kenji Osafune

**Affiliations:** 1 Center for iPS Cell Research and Application (CiRA), Kyoto University, 53 Kawahara-cho, Shogoin, Sakyo-ku, Kyoto 606-8507, Japan

**Keywords:** ESC, iPSC, differentiation, pancreas, pancreatic bud, pancreatic endoderm, NKX6.1, ROCK, non-muscle myosin II, cytoskeleton

## Abstract

There has been increasing success with the generation of pancreatic cells from human induced pluripotent stem cells (hiPSCs); however, the molecular mechanisms of the differentiation remain elusive. The purpose of this study was to reveal novel molecular mechanisms for differentiation to PDX1^+^NKX6.1^+^ pancreatic endoderm cells, which are pancreatic committed progenitor cells. PDX1^+^ posterior foregut cells differentiated from hiPSCs failed to differentiate into pancreatic endoderm cells at low cell density, but Rho-associated kinase (ROCK) or non-muscle myosin II (NM II) inhibitors rescued the differentiation potential. Consistently, the expression of phosphorylated myosin light chain 2 and NM IIA was downregulated in aggregation culture. Notably, the soluble factors we tested were substantially effective only with ROCK-NM II inhibition. The PDX1^+^NKX6.1^+^ cells induced with NM II inhibitors were successfully engrafted and maturated *in vivo*. Taken together, these results suggest that NM IIs play inhibitory roles for the differentiation of hiPSCs to pancreatic endoderm cells.

## Highlights

• ROCK and NM II inhibition facilitates the induction of pancreatic endoderm cells
• Aggregation cultures downregulate ROCK and NM II signaling molecules
• Induced pancreatic cells differentiate into functional endocrine cells *in vivo*
• We propose ROCK-NM II signaling suppresses pancreatic endoderm formation *in vitro*

## Graphical Abstract

**Figure:**
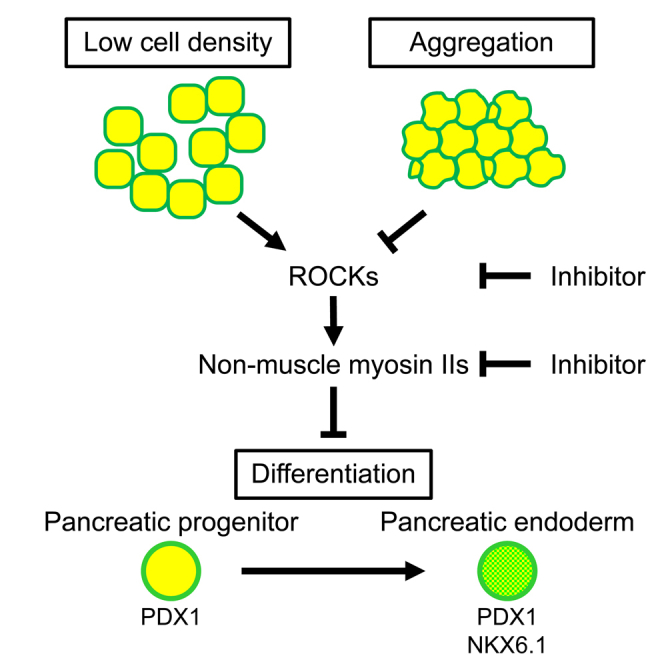

## Teaser

Toyoda and colleagues show that ROCK or NM II inhibitors facilitate differentiation of PDX1^+^ posterior foregut cells into PDX1^+^NKX6.1^+^ pancreatic endoderm cells in hiPSC cultures. Consistently, NM II expression was elevated at low cell density, but upon aggregation, it was downregulated and associated with NKX6.1^+^ cell induction. The results provide mechanistic insight into the benefit of three-dimensional cultures for directed differentiation.

## Introduction

Pancreatic cells generated from pluripotent stem cells, such as human embryonic stem cells (hESCs) and induced pluripotent stem cells (hiPSCs), are considered a promising cell source for regenerative therapies. Recent advances in regenerative medicine research have demonstrated that guided differentiation can recapitulate normal developmental stages and enables the generation of pancreatic cells, including mature type pancreatic beta cells *in vitro* (Pagliuca et al., 2014, Rezania et al., 2014). Among these stages, the cell type in pancreatic bud formation is crucial, since these cells are the earliest stage of pancreatic endoderm cells and considered committed to differentiate into only pancreatic lineages (Kelly et al., 2011, Rezania et al., 2013). Several reports have shown the efficient induction of PDX1^+^NKX6.1^+^ pancreatic endoderm cells, which correspond to cells at the stages from pancreatic bud to branched epithelia, from hESCs/iPSCs (Nostro et al., 2015, Pagliuca et al., 2014, Rezania et al., 2014, Russ et al., 2015, Toyoda et al., 2015). However, the molecular mechanisms regulating this differentiation remain elusive, which potentially causes unstable manipulation of the cells and contamination of other cell types, thus hampering basic research and clinical application.

The cellular morphology and physical microenvironment dramatically change during differentiation. In pancreas development, the first step of organogenesis is the formation of the pancreatic bud (Villasenor et al., 2010). A pre-pancreatic region at gut tube endoderm composes a single layer of epithelial cells that express *PDX1*. With the progress in pancreatic specification, the epithelium rapidly thickens to form an aggregation of cells called the pancreatic bud, which concomitantly express the pancreas-specific markers *NKX6.1* and *PTF1A*. This process is closely linked to changes in cell shape from cuboidal to columnar and squamous (Villasenor et al., 2010) and changes in the microenvironment, such as cell-to-cell interactions, matrix stiffness, and cell polarity. It is possible that these physical changes lead to modifications in intracellular signaling, resulting in modulation of the propensity for differentiation into pancreatic lineages (Cortijo et al., 2012, Kesavan et al., 2009). Supporting this idea, we previously showed that high cell density or aggregation promotes the differentiation of PDX1^+^ posterior foregut cells to the earliest stage of PDX1^+^NKX6.1^+^ pancreatic endoderm cells in hESC/iPSC differentiation cultures (Toyoda et al., 2015). In either high-cell-density or aggregation culture, cells are condensed, suggesting a different physical environment from that in low-cell-density cultures. In many cell types, it is suggested that manipulation of the cellular morphology or physical microenvironment affects the cell state and differentiation propensity via cytoskeletal changes (Connelly et al., 2010, Maharam et al., 2015, Sun et al., 2014). More specifically, genetic and chemical modifications of cytoskeleton regulators disrupt progenitor cell proliferation, organ size, and composition of the differentiated cells in the developing pancreas (Kesavan et al., 2009, Petzold et al., 2013, Shih et al., 2016). Thus, proper cytoskeletal regulation is required for normal pancreatic organogenesis; however, the roles of the cytoskeleton in the formation of pancreatic endoderm remain unclear.

In the current study, we tested the hypothesis that molecules related to the cytoskeleton regulate the differentiation of hESC/iPSC-derived PDX1^+^ posterior foregut cells to PDX1^+^NKX6.1^+^ pancreatic endoderm cells. We found that the expression of non-muscle myosin IIs (NM IIs) is downregulated in aggregation cultures and that chemical inhibition of Rho-associated kinases (ROCKs) and NM IIs promotes differentiation to pancreatic endoderm cells, mimicking the effect of aggregation cultures.

## Results

### ROCK-NM II Inhibitors Facilitate the Differentiation of Posterior Foregut to Pancreatic Endoderm Cells

**Figure 1:**
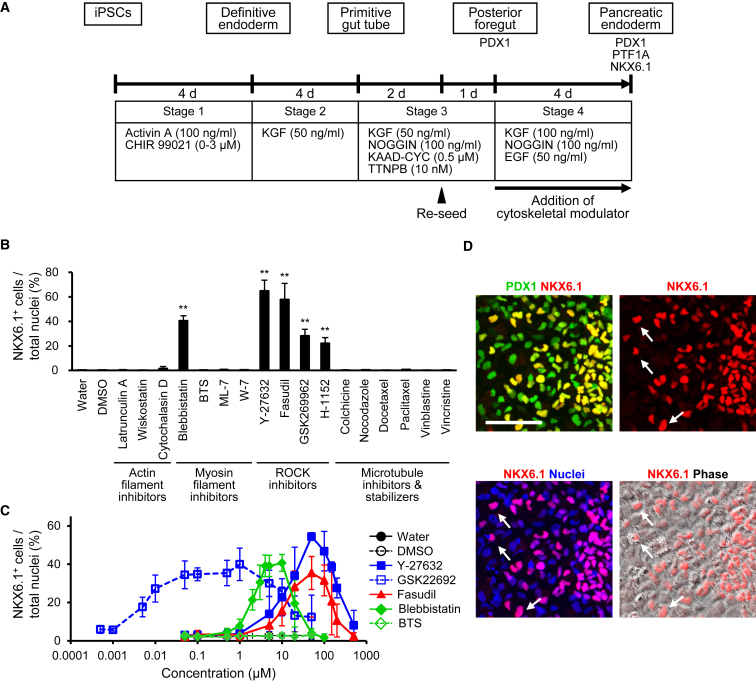
ROCK Signaling Inhibitors Promote PDX1^+^NKX6.1^+^ Cell Induction from PDX1^+^ Posterior Foregut Cells (A) A schematic diagram of the procedure used to differentiate pancreatic endoderm cells from hiPSCs. (B and C) Quantification of the proportion of NKX6.1^+^ cells by an image analyzer. Note that water, DMSO, and BTS treatment did not induce NKX6.1^+^ cells at any concentration in (C). (D) PDX1^+^NKX6.1^+^ cells were localized in sparse areas (arrows) in Y-27632-treated cells in monolayer culture. Data are presented as the mean ± SD from three independent experiments in (B) and (C). ^∗∗^p < 0.01 versus water. Scale bar, 100 μm. See also Figures S1 and S2.

PDX1^+^ posterior foregut cells re-seeded at low cell densities (1.6 × 10^5^ cells/cm^2^) were treated with three soluble factors: KGF, NOGGIN, and EGF (Figure 1A). As expected, this cell density was too low to induce NKX6.1^+^ cells even after 4 days of treatment (water- or DMSO-treated group in Figure 1B) (Toyoda et al., 2015). To investigate whether modulation of the cytoskeleton promotes differentiation to pancreatic endoderm cells, we treated the PDX1^+^ cells with inhibitors of actin and myosin II filaments and ROCK and with microtubule inhibitors/stabilizers in addition to the aforementioned three factors for 3 days. We found one myosin II inhibitor (Blebbistatin) and four ROCK inhibitors (Y-27632, Fasudil, GSK269962, and H-1152) increased the proportion of NKX6.1^+^ cells, while a muscle-type myosin II inhibitor, BTS, and a low dose of Y-27632, which induced actin disassembly, failed to induce NKX6.1^+^ cells (Figures 1B, S1, S2A, and S2B). These results suggest that the signaling of ROCKs and downstream NM IIs regulates differentiation into NKX6.1^+^ cells, although we cannot exclude the possibility that the inhibitors used in this study may target molecules other than ROCK-NM II (Figure S2A). GSK269962, which has the highest affinity for ROCK (half-maximal inhibitory concentration [IC_50_] for ROCK1 of GSK269962, Y-27632, and Fasudil: 1.6, 150, and 300 nM, respectively), had the most potent inducing activity (Figure 1C) (Doe et al., 2007, Jacobs et al., 2006), supporting the involvement of ROCK-NM II signaling. Cells treated with ROCK-NM II inhibitors tended to be small, but not all small cells were NKX6.1^+^ and large NKX6.1^+^ cells were also observed (Figures 1D and S1 and data not shown). Notably, NKX6.1^+^ cells were observed in a relatively sparse area as well as a high-density area (Figure 1D), suggesting that cell aggregation is not necessary for NKX6.1^+^ cell induction with treatment of ROCK-NM II inhibitors.

### NKX6.1^+^ Cells Induced with ROCK-NM II Inhibitors Show Developmental Potential Consistent with Pancreatic Endoderm Cells

**Figure 2:**
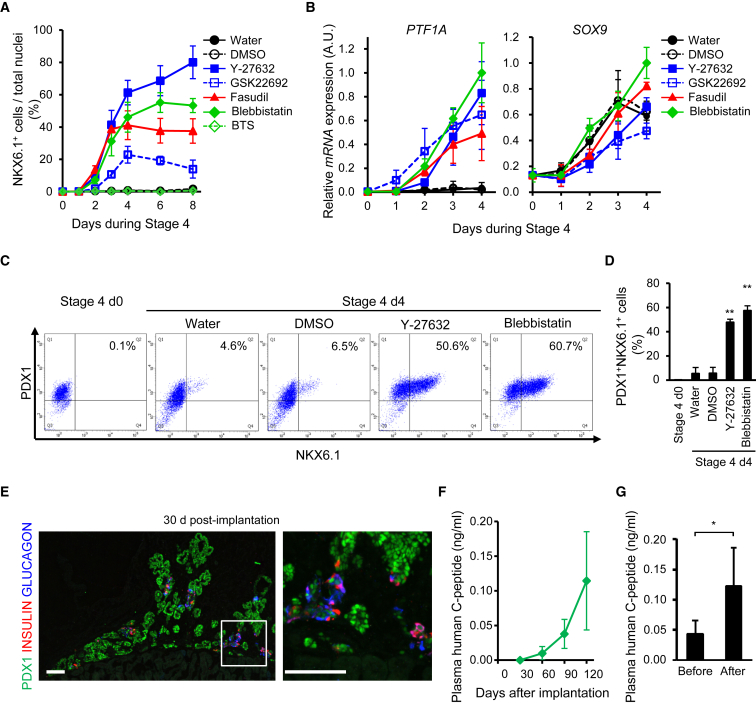
NKX6.1^+^ Cells Induced with ROCK-NM II Inhibitors Show Developmental Potential Consistent with Pancreatic Endoderm Cells (A) Time course for NKX6.1^+^ cell induction by ROCK-NM II inhibitors was analyzed by an image analyzer. Note that water, DMSO, and BTS treatment did not induce NKX6.1^+^ cells at any concentration. (B) The mRNA expression of a pancreatic endoderm marker, *PTF1A*, and a ductal progenitor marker, *SOX9*. (C and D) The proportion of PDX1^+^NKX6.1^+^ cells was analyzed by flow cytometry. Representative dot plots (C) and quantification of the induction efficiency (D). (E) PDX1^+^NKX6.1^+^ cells induced with Blebbistatin developed into branched pancreatic epithelia *in vivo*. A representative cryosection image of grafts 30 days after implantation from three independent experiments was stained for the indicated markers. The right panel shows a magnified image of the boxed area in the left panel. (F and G) Plasma human C-peptide levels in host mice at various time points after implantation (F) and before and after glucose injection on days 120–151 after implantation (G). Data are presented as the mean ± SD from three independent experiments in (A, B, and D) and as the mean ± SE of three mice from two independent cohorts of implantation experiments in (F) and (G). ^∗^p < 0.05. ^∗∗^p < 0.01 versus water. Scale bars, 100 μm. See also Figures S2 and S3.

Next, we validated the developmental potential of NKX6.1^+^ cells induced with ROCK-NM II inhibitors. The mRNA expression of *PTF1A*, a pancreatic endoderm cell marker, was associated with an increase in the proportion of NKX6.1^+^ cells in ROCK-NM II inhibitor-treated cells, whereas the expression of a ductal multipotent progenitor marker, *SOX9*, was increased in all groups with time (Figures 2A and 2B). Flow cytometric analysis demonstrated that all NKX6.1^+^ cells induced by ROCK-NM II inhibitors co-expressed PDX1 (Figures 2C and 2D). The positive effects of ROCK-NM II inhibition on differentiation into NKX6.1^+^ cells were reproduced in hESC line KhES-3 and hiPSC line Ff-I01 (Figure S2C). Moreover, the cells treated with Y-27632 or Blebbistatin at stage 4 were able to form PDX1^+^ tubular structures that possess INSULIN^+^ and GLUCAGON^+^ endocrine cells, which is reminiscent of human embryonic pancreatic epithelia, 30 days after implantation into the renal subcapsule of immunodeficient mice (Figures 2E and S3A). Plasma human C-peptide levels, which indicate human insulin secretion, gradually increased over time and eventually responded to changes in blood glucose levels in mice implanted with Blebbistatin-treated cells (Figures 2F, 2G, and S3B–S3D). These results suggest that the NKX6.1^+^ cells induced with ROCK-NM II inhibitors are pancreatic endoderm cells that have the developmental potential to differentiate into pancreatic epithelia and mature into β cells *in vivo*.

### NM II Signaling Molecules Are Downregulated in Aggregation Culture

**Figure 3:**
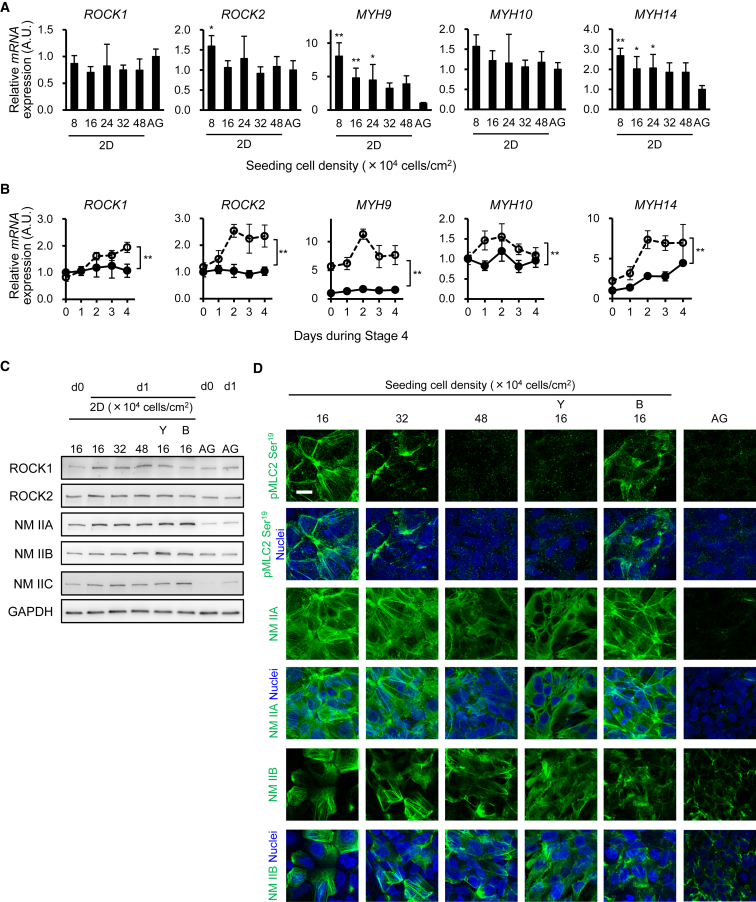
ROCK-NM II Signaling Is Downregulated in Aggregation Cultures (A and B) PDX1^+^ posterior foregut cells were re-seeded either for monolayer cultures (2D) or to form cellular aggregates (3 × 10^4^ cells/aggregate, AG). The next day, the cells were exposed to stage 4 treatment without ROCK-NM II inhibitors. The mRNA expression of genes encoding ROCKs and NM IIs in the cells on stage 4 day 0 (A) and its time course in AG (black circle, solid line) and 2D (1.6 × 10^5^ cells/cm^2^, white circle, dotted line) (B). (C and D) Representative images of the expression levels of ROCK and NM II proteins on stage 4 days 0 and 1 (C) and ROCK downstream molecules on stage 4 day 1 (D) of three independent experiments. Data are presented as the mean ± SD from four independent experiments in (A) and (B). ^∗^p < 0.05, ^∗∗^p < 0.01 versus AG. Y, Y-27632 (50 μM). B, Blebbistatin (5 μM). Scale bar, 20 μm. See also Figure S4.

ROCK-NM II inhibition facilitates differentiation into pancreatic endoderm cells without high-cell-density or aggregation cultures. To examine whether ROCK-NM II signaling is inhibited in high-cell-density or aggregation cultures, we analyzed the mRNA expression of genes encoding ROCK1 and ROCK2 (*ROCK1* and *ROCK2*) and NM IIA, NM IIB, and NM IIC (*MYH9*, *MYH10*, and *MYH14*) in monolayer-cultured cells at different cell densities (8–48 × 10^4^ cells/cm^2^) and in cellular aggregates on stage 4 day 0. We found a tendency for the mRNA expression of *MYH9* and *MYH14* to decrease as the cell density increased (Figure 3A). Notably, the mRNA expression of *MYH9* and *MYH14* was lowest in the cellular aggregates. Interestingly, the mRNA expression of all five genes was significantly lower in the cellular aggregates than in low-cell-density monolayer cultures at stage 4 (Figure 3B). Consistent with these findings, the protein levels of NM IIA and NM IIC, as evaluated by western blotting, were lowest in the cellular aggregates (Figures 3C and S4A), and the levels of phosphorylated myosin light chain 2 (pMLC2), which indicates ROCK activity (Amano et al., 1996), and NM IIA, as evaluated by immunostaining, were weaker in high-cell-density and aggregation cultures than in low-cell-density cultures (Figure 3D). The difference in the results of NM IIA expression with high-cell-density cultures between western blotting and immunostaining is possibly due to the different sensitivity and targets of each method. Western blotting evenly detects all cellular NM IIA molecules, whereas immunostaining emphasizes accumulated NM IIA molecules such as polymeric fibers compared with monomers. Taken together, these results suggest that signaling related to ROCK-NM II is suppressed multiple ways by aggregation cultures.

### Differentiation Mechanisms by which ROCK-NM II Inhibitors Induce Pancreatic Endoderm Cells Mimic Aggregation Effects

**Figure 4:**
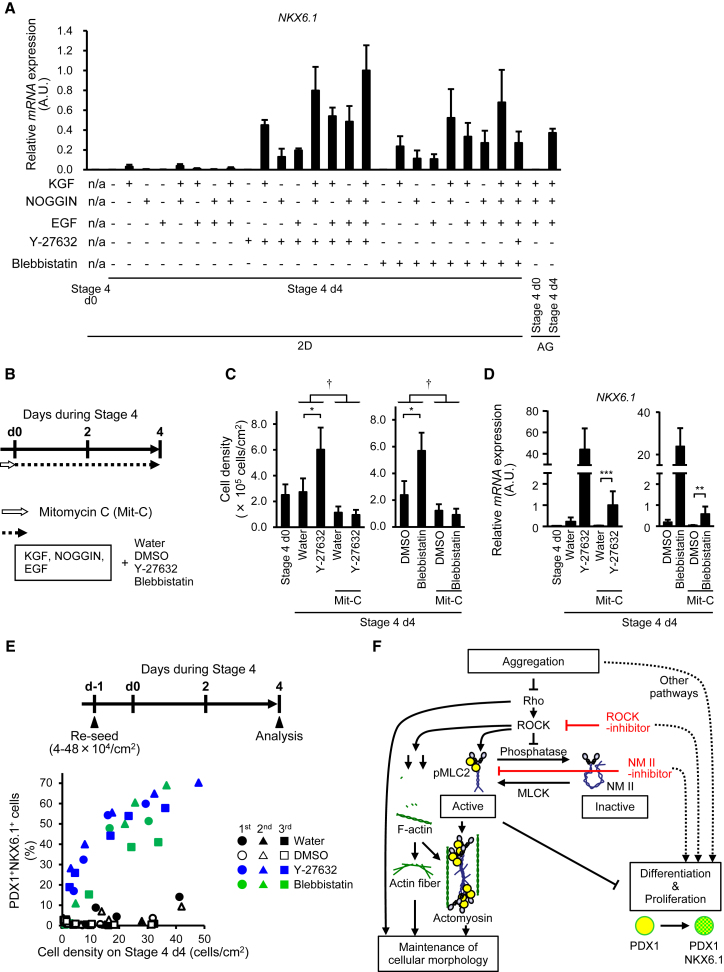
ROCK-NM II Inhibitors Induce NKX6.1^+^ Cells via Proliferation-Independent Mechanisms (A) mRNA expression of *NKX6.1* in cells treated with various combinations of soluble factors (100 ng/mL KGF, 100 ng/mL NOGGIN, and 50 ng/mL EGF) and ROCK-NM II inhibitors (50 μM Y-27632 and 5 μM Blebbistatin) for 4 days of stage 4 monolayer culture. (B) A schematic diagram of the procedures for (C) and (D). PDX1^+^ posterior foregut cells were pre-treated with mitomycin C (47 μM, 2 hr) to inhibit proliferation before pancreatic endoderm cell induction. (C and D) Cell density (C) and mRNA expression of *NKX6.1* (D) in cells after 4 days of pancreatic endoderm induction. (E) PDX1^+^ posterior foregut cells were re-seeded at various cell densities (4–48 × 10^4^ cells/cm^2^). The next day, cells were cultured in stage 4 medium with or without Y-27632 (50 μM) or Blebbistatin (5 μM). After 4-day culture, the relationship between cell density and the proportion of PDX1^+^NKX6.1^+^ cells was analyzed by flow cytometry. The scatterplot indicates data from three independent experiments. (F) A model showing the regulation of pancreatic endoderm cell differentiation. Note that the involvement of the ROCK-NM II pathway in the maintenance of cellular morphology was not analyzed in this study. Data are presented as the mean ± SD from three independent experiments in (A), (C), and (D). ^∗^p < 0.05, ^∗∗^p < 0.01, ^∗∗∗^p < 0.001, versus water or DMSO. ^†^p < 0.05, versus mitomycin C. n/a, not applicable.

We previously found that the signals induced by cell aggregation cultures for pancreatic endoderm cell induction are different from those induced by soluble factors (KGF, NOGGIN, and EGF) (Toyoda et al., 2015). The combination of cell aggregation cultures with any one of these soluble factors upregulated *NKX6.1* expression. Similar to the effects of cell aggregation, a combination of ROCK-NM II inhibitors and one soluble factor also increased the expression of *NKX6.1* (Figure 4A). These results suggest that the signals regulated by ROCK-NM II inhibition are independent of those induced by the three aforementioned factors.

Next, we examined whether ROCK-NM II inhibition works through high-cell-density or aggregation effects caused by cell proliferation. The expression of *NKX6.1* was increased by Y-27632 or Blebbistatin treatment under the inhibition of cell proliferation by pre-treatment with a mitotic inactivator, mitomycin C (Figures 4B–4D and S4B). Y-27632 or Blebbistatin treatment did not prevent apoptosis, as evaluated by immunostaining against an apoptotic marker, cleaved caspase-3 (Figures S4C and S4D). In addition, we analyzed the relationship between the proportion of PDX1^+^NKX6.1^+^ cells and cell density on stage 4 day 4. Scatterplots demonstrated that the efficiency of PDX1^+^NKX6.1^+^ cell induction was higher in ROCK-NM II inhibitor-treated cells than the corresponding controls (water- or DMSO-treated cells) at similar cell densities (Figure 4E). These findings support the idea that ROCK-NM II inhibition induces pancreatic endoderm cells by mechanisms that mimic aggregation effects but not by increasing the cell number, resulting in high-cell-density or aggregation effects.

## Discussion

We previously reported that high-cell-density or aggregation culture is beneficial for the induction of PDX1^+^ posterior foregut cells to PDX1^+^NKX6.1^+^ pancreatic endoderm cells, however, the molecular mechanisms were undetermined (Toyoda et al., 2015). In the present study, we found that ROCK-NM II inhibition facilitated PDX1^+^NKX6.1^+^ cell induction even in low-cell-density cultures. In addition, ROCK-NM II inhibition effectively induced differentiation when combined with known soluble factors (KGF, EGF, and NOGGIN), which indicates a similar mechanism to that of aggregation cultures. These results suggest that ROCK-NM II inhibition is an independent factor for pancreatic endoderm cell induction, leading us to propose that ROCK-NM II inhibition may be one of the mechanisms underlying the effective pancreatic endoderm cell induction seen in aggregation cultures.

Two models can explain the mechanisms with which ROCK-NM II inhibition induces pancreatic endoderm cells. In one model, ROCK-NM II inhibition increases the cell number, resulting in the high-cell-density or aggregation condition that is favorable for differentiation. In the other model, ROCK-NM II inhibition directly regulates differentiation signals. *NKX6.1* mRNA expression was induced by Y-27632 or Blebbistatin under the inhibition of cell proliferation or without the disruption of apoptosis, suggesting that *NKX6.1* induction is at least in part via a mechanism independent of cell density. Consistently, ROCK-NM II inhibition had a higher induction efficiency of PDX1^+^NKX6.1^+^ cells than corresponding controls with the same cell densities, including cell densities at lower ranges. Based on these observations, it is likely that ROCK-NM II inhibition directly regulates signals mimicking those induced by aggregation cultures.

The priming of cell differentiation and commitment into certain cell types are coordinated by the balance of inducing and inhibiting signals. We found that cells in high-cell-density or aggregation cultures, which favor differentiation to pancreatic endoderm cells, had lower ROCK activity than cells in low-cell-density cultures, and the expression of NM IIA mRNA and protein was lowest in aggregation cultures. Therefore, it is reasonable to assume that NM IIA suppresses differentiation into pancreatic endoderm cells. We also found that low-cell-density cultures increased ROCK-NM II mRNA expression with time. Based on these findings, we propose that binding to substances other than the surface of surrounding cells may induce cytoskeletal modifications, including the upregulation of ROCK-NM II, which in turn suppresses NKX6.1 expression. Three-dimensional cultures are often used to mimic the *in vivo* microenvironment, and the benefits of such cultures have been reported in other cell types (Ogawa et al., 2013, Schyschka et al., 2013). Our findings shed light on the molecular mechanisms that give three-dimensional cultures advantages over two-dimensional cultures.

Although the detailed mechanisms by which NM IIA suppresses differentiation into pancreatic endoderm cells remain to be elucidated, we assume that NM II-associated proteins or downstream molecules may act as transcriptional regulators that repress *NKX6.1* gene expression either directly or indirectly. Some reports have described the detailed molecular mechanisms by which alterations in cytoskeletal structures regulate cellular differentiation. In mouse dedifferentiated fat cells, the disruption of actin fibers drives adipogenic differentiation through an increase in interactions between monomeric G-actin and megakaryoblastic leukemia 1 protein (Nobusue et al., 2014). In mesenchymal stem cells, information on the cell shape and soft extracellular matrix environment is transduced by two transcriptional factors, Yes-associated protein (YAP) and transcriptional coactivator with PDZ-binding motif (TAZ), which enables adipogenic differentiation (Dupont et al., 2011). Although G-actin binding protein and the YAP/TAZ system are attractive candidate mediators, we found that PDX1^+^NKX6.1^+^ cell induction is specifically regulated by the inhibition of ROCKs and NM IIs, and not by any actin fiber or microtubule disassembling reagents. In addition, we could not find any association between cell shape or size with NKX6.1^+^ cell induction when cells were treated with cytoskeletal inhibitors. Therefore, the involvement of G-actin binding protein or the YAP/TAZ system is unlikely. A previous study reported that Fyn kinase downregulates ROCK-NM II during oligodendrocyte morphological differentiation in mice (Wang et al., 2012). Another report demonstrated that an NM II isoform, MYH9, interacts with nuclear protein Thy28/Thyn1, and that this complex binds to *Pax5* 1A promoter to suppress *Pax5* expression in chicken B cells (Fujita et al., 2015). Similarly, the elucidation of regulatory molecules or binding partners for NM II may provide new transcriptional regulating mechanisms of pancreatic development.

The efficiency of NKX6.1^+^ cell induction tended to be high at high cell density, even in ROCK-NM II inhibitor-treated cells, suggesting ROCK-NM II independent effects on cell density. One possible explanation for these effects is that NKX6.1^+^ cells may have high proliferation capacity at high cell density. Another explanation considers high-cell-density signals through both ROCK-NM II and other signaling pathways.

In summary, treatment with ROCK-NM II inhibitors or aggregation cultures that downregulate ROCK-NM II signaling facilitates the differentiation of hiPSC-derived PDX1^+^ posterior foregut cells to PDX1^+^NKX6.1^+^ pancreatic endoderm cells. We propose that insufficient cell density may induce NM II activation, suppressing differentiation to pancreatic endoderm cells (Figure 4F). In this study, we demonstrated that ROCK-NM II signals associated with physical contexts regulate the differentiation of hESCs/iPSCs into pancreatic linages. Our findings should contribute to the development of stable cell supplies for hESC/iPSC-derived pancreatic cells.

## Experimental Procedures

### hESC/iPSC Culture and Differentiation

An hESC line KhES-3 and two hiPSC lines 585A1 and Ff-I01 were maintained as described previously (Toyoda et al., 2015). Experiments with hESCs/iPSCs were approved by the ethics committee of the Department of Medicine and Graduate School of Medicine, Kyoto University. Cells were directed into key stages of pancreatic development as described previously with modifications (Figure 1A; Supplemental Experimental Procedures).

### Immunostaining

Cells were fixed with 4% paraformaldehyde (PFA) for 20 min at 4°C. The implanted grafts were fixed with 4% PFA for 1–2 days at 4°C, then the samples were equilibrated in a 10%–30% sucrose solution, mounted, and frozen. The frozen blocks were sectioned at 10 μm. Immunostaining was performed as described previously (Toyoda et al., 2015). The primary antibodies used are detailed in Table S1. Filamentous-actin (F-actin) was stained with Acti-stain phalloidin (Cytoskeletal, Denver, CO).

### Flow Cytometry

Cells were dissociated into single cells with 0.25% trypsin–EDTA treatment, fixed, permeabilized and blocked with a BD Cytofix/Cytoperm Kit (Becton Dickinson, Franklin Lakes, NJ). Then, the cells were stained with the antibodies as detailed in Table S1. Stained undifferentiated hiPSCs and stage 4 day 0 cells were used as negative controls for gating.

### Implantation Experiments

All animal experiments were performed in accordance with the Guidelines for Animal Experiments of Kyoto University. Male 7- to 13-week-old NOD.CB17-Prkdcscid/J mice were anesthetized with inhalable isoflurane and received implants of hiPSC-derived cell aggregates after stage 4 under a kidney subcapsule. After the cells on stage 4 day 4 formed aggregates (3 × 10^4^ cells/aggregate), they were cultured in stage 4 medium with or without an ALK5 inhibitor for an additional 2 days before implantation. All metabolic analyses were performed in conscious and restrained animals.

## Author Contributions

T.T. wrote the first draft of the manuscript. T.T., A.K., and K.O. contributed to the manuscript edits and revisions. T.T. designed, directed, and interpreted the experiments. T.T., A.K., H.T., T.A., A.M., Y.H., M.N., and A.W. performed the experiments. K.O. supervised the study.

## Supplemental Information

Document S1. Supplemental Experimental Procedures, Figures S1–S4, and Tables S1 and S2

Document S2. Article plus Supplemental Information

## References

[bib1] Amano M., Ito M., Kimura K., Fukata Y., Chihara K., Nakano T., Matsuura Y., Kaibuchi K. (1996). Phosphorylation and activation of myosin by Rho-associated kinase (Rho-kinase). J. Biol. Chem..

[bib2] Connelly J.T., Gautrot J.E., Trappmann B., Tan D.W., Donati G., Huck W.T., Watt F.M. (2010). Actin and serum response factor transduce physical cues from the microenvironment to regulate epidermal stem cell fate decisions. Nat. Cell Biol..

[bib3] Cortijo C., Gouzi M., Tissir F., Grapin-Botton A. (2012). Planar cell polarity controls pancreatic beta cell differentiation and glucose homeostasis. Cell Rep..

[bib4] Doe C., Bentley R., Behm D.J., Lafferty R., Stavenger R., Jung D., Bamford M., Panchal T., Grygielko E., Wright L.L. (2007). Novel Rho kinase inhibitors with anti-inflammatory and vasodilatory activities. J. Pharmacol. Exp. Ther..

[bib5] Dupont S., Morsut L., Aragona M., Enzo E., Giulitti S., Cordenonsi M., Zanconato F., Le Digabel J., Forcato M., Bicciato S. (2011). Role of YAP/TAZ in mechanotransduction. Nature.

[bib6] Fujita T., Kitaura F., Fujii H. (2015). A critical role of the Thy28-MYH9 axis in B cell-specific expression of the Pax5 gene in chicken B cells. PLoS One.

[bib7] Jacobs M., Hayakawa K., Swenson L., Bellon S., Fleming M., Taslimi P., Doran J. (2006). The structure of dimeric ROCK I reveals the mechanism for ligand selectivity. J. Biol. Chem..

[bib8] Kelly O.G., Chan M.Y., Martinson L.A., Kadoya K., Ostertag T.M., Ross K.G., Richardson M., Carpenter M.K., D'Amour K.A., Kroon E. (2011). Cell-surface markers for the isolation of pancreatic cell types derived from human embryonic stem cells. Nat. Biotechnol..

[bib9] Kesavan G., Sand F.W., Greiner T.U., Johansson J.K., Kobberup S., Wu X., Brakebusch C., Semb H. (2009). Cdc42-mediated tubulogenesis controls cell specification. Cell.

[bib10] Maharam E., Yaport M., Villanueva N.L., Akinyibi T., Laudier D., He Z., Leong D.J., Sun H.B. (2015). Rho/Rock signal transduction pathway is required for MSC tenogenic differentiation. Bone Res..

[bib11] Nobusue H., Onishi N., Shimizu T., Sugihara E., Oki Y., Sumikawa Y., Chiyoda T., Akashi K., Saya H., Kano K. (2014). Regulation of MKL1 via actin cytoskeleton dynamics drives adipocyte differentiation. Nat. Commun..

[bib12] Nostro M.C., Sarangi F., Yang C., Holland A., Elefanty A.G., Stanley E.G., Greiner D.L., Keller G. (2015). Efficient generation of NKX6-1+ pancreatic progenitors from multiple human pluripotent stem cell lines. Stem Cell Reports.

[bib13] Ogawa S., Surapisitchat J., Virtanen C., Ogawa M., Niapour M., Sugamori K.S., Wang S., Tamblyn L., Guillemette C., Hoffmann E. (2013). Three-dimensional culture and cAMP signaling promote the maturation of human pluripotent stem cell-derived hepatocytes. Development.

[bib14] Pagliuca F.W., Millman J.R., Gurtler M., Segel M., Van Dervort A., Ryu J.H., Peterson Q.P., Greiner D., Melton D.A. (2014). Generation of functional human pancreatic beta cells in vitro. Cell.

[bib15] Petzold K.M., Naumann H., Spagnoli F.M. (2013). Rho signalling restriction by the RhoGAP Stard13 integrates growth and morphogenesis in the pancreas. Development.

[bib16] Rezania A., Bruin J.E., Xu J., Narayan K., Fox J.K., O'Neil J.J., Kieffer T.J. (2013). Enrichment of human embryonic stem cell-derived NKX6.1-expressing pancreatic progenitor cells accelerates the maturation of insulin-secreting cells in vivo. Stem Cells.

[bib17] Rezania A., Bruin J.E., Arora P., Rubin A., Batushansky I., Asadi A., O'Dwyer S., Quiskamp N., Mojibian M., Albrecht T. (2014). Reversal of diabetes with insulin-producing cells derived in vitro from human pluripotent stem cells. Nat. Biotechnol..

[bib18] Russ H.A., Parent A.V., Ringler J.J., Hennings T.G., Nair G.G., Shveygert M., Guo T., Puri S., Haataja L., Cirulli V. (2015). Controlled induction of human pancreatic progenitors produces functional beta-like cells in vitro. EMBO J..

[bib19] Schyschka L., Sanchez J.J., Wang Z., Burkhardt B., Muller-Vieira U., Zeilinger K., Bachmann A., Nadalin S., Damm G., Nussler A.K. (2013). Hepatic 3D cultures but not 2D cultures preserve specific transporter activity for acetaminophen-induced hepatotoxicity. Arch. Toxicol..

[bib20] Shih H.P., Panlasigui D., Cirulli V., Sander M. (2016). ECM signaling regulates collective cellular dynamics to control pancreas branching morphogenesis. Cell Rep..

[bib21] Sun Y., Yong K.M., Villa-Diaz L.G., Zhang X., Chen W., Philson R., Weng S., Xu H., Krebsbach P.H., Fu J. (2014). Hippo/YAP-mediated rigidity-dependent motor neuron differentiation of human pluripotent stem cells. Nat. Mater..

[bib22] Toyoda T., Mae S., Tanaka H., Kondo Y., Funato M., Hosokawa Y., Sudo T., Kawaguchi Y., Osafune K. (2015). Cell aggregation optimizes the differentiation of human ESCs and iPSCs into pancreatic bud-like progenitor cells. Stem Cell Res..

[bib23] Villasenor A., Chong D.C., Henkemeyer M., Cleaver O. (2010). Epithelial dynamics of pancreatic branching morphogenesis. Development.

[bib24] Wang H., Rusielewicz T., Tewari A., Leitman E.M., Einheber S., Melendez-Vasquez C.V. (2012). Myosin II is a negative regulator of oligodendrocyte morphological differentiation. J. Neurosci. Res..

