# Supplementary material for: Rho-Associated Kinases and Non-muscle Myosin IIs Inhibit the Differentiation of Human iPSCs to Pancreatic Endoderm
Source: Stem Cell Reports. 2017 Aug 8;9(2):419–28. doi: 10.1016/j.stemcr.2017.07.005 (PMC5550204; doi:10.1016/j.stemcr.2017.07.005)
Supplement: Document S1. Supplemental Experimental Procedures, Figures S1–S4, and Tables S1 and S2 [file mmc1.pdf]

Stem Cell Reports, Volume *9*

Supplemental Information

Rho-Associated Kinases and Non-muscle Myosin IIs Inhibit the Differentiation of Human iPSCs to Pancreatic Endoderm

Taro Toyoda, Azuma Kimura, Hiromi Tanaka, Tomonaga Ameku, Atsushi Mima, Yurie Hirose, Masahiro Nakamura, Akira Watanabe, and Kenji Osafune

Figure S1

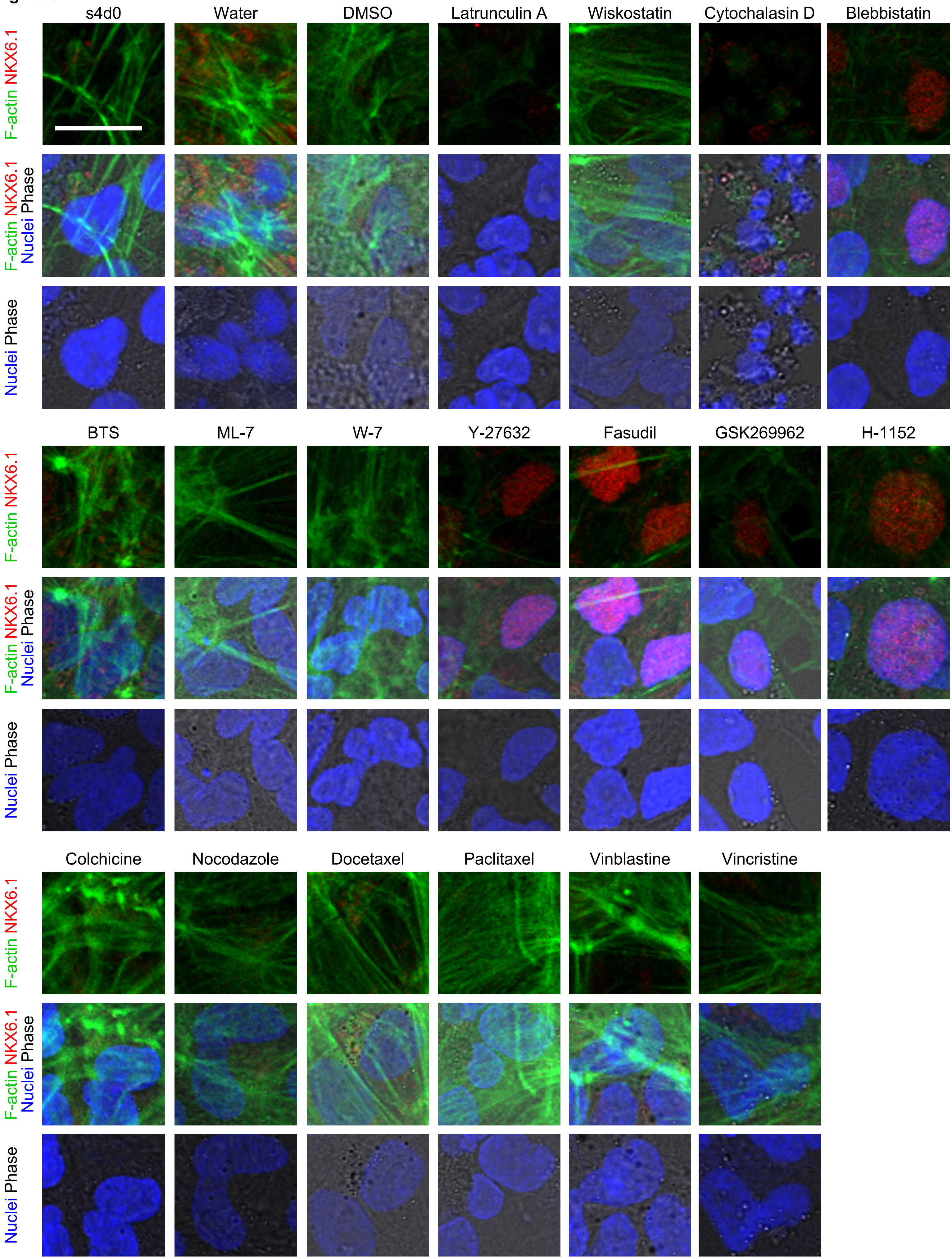
s4d0
Water
DMSO
Latrunculin A
Wiskostatin
Cytochalasin D
Blebbistatin
BTS
ML-7
W-7
Y-27632
Fasudil
GSK269962
H-1152
Colchicine
Nocodazole
Docetaxel
Paclitaxel
Vinblastine
Vincristine
F-actin NKX6.1
F-actin NKX6.1 Nuclei Phase
Nuclei Phase

Figure S2

A

Name	Main targets	Other targets
Latrunculin A	Monomeric actin	
Wiskostatin	N-WASP	
Cytochalasin D	Filamentous actin	
Blebbistatin	Myosin II	
BTS	Skeletal muscle myosin II	
ML-7	MLCK	
W-7	Calmodulin, PDE1 and MLCK	
Y-27632	ROCK1	PKC, PKA, MLCK and PRK2
Fasudil	PKA and ROCK	
GSK269962	ROCK1 and ROCK2	
H-1152	ROCK2	CAMKII, PKG, Aurora A, PKA, PKC and MLCK
Colchicine	Tubulin	
Nocodazole	Tubulin	
Docetaxel	Microtubule	
Paclitaxel	Microtubule	
Vinblastine	Tubulin	
Vincristine	Tubulin	

B

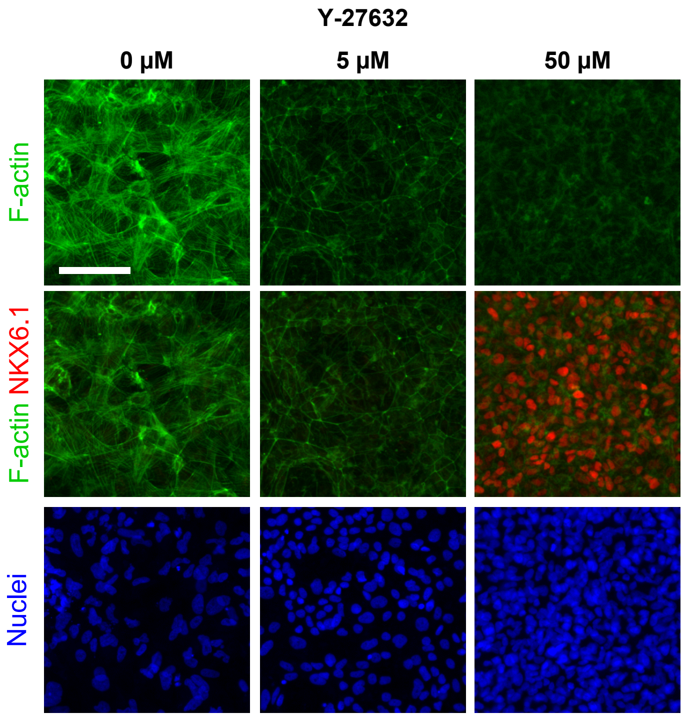

C

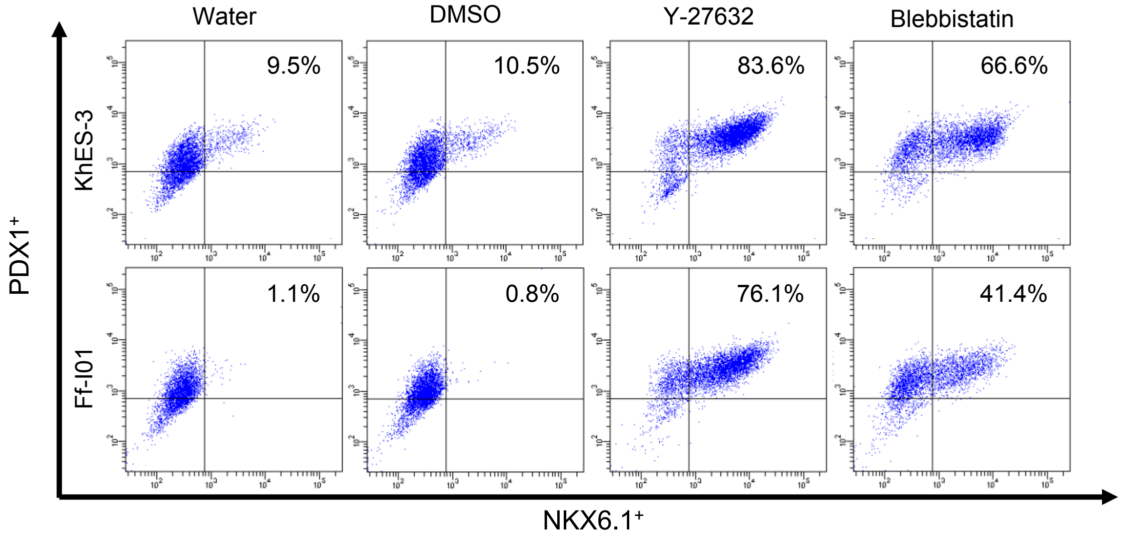

Figure S3

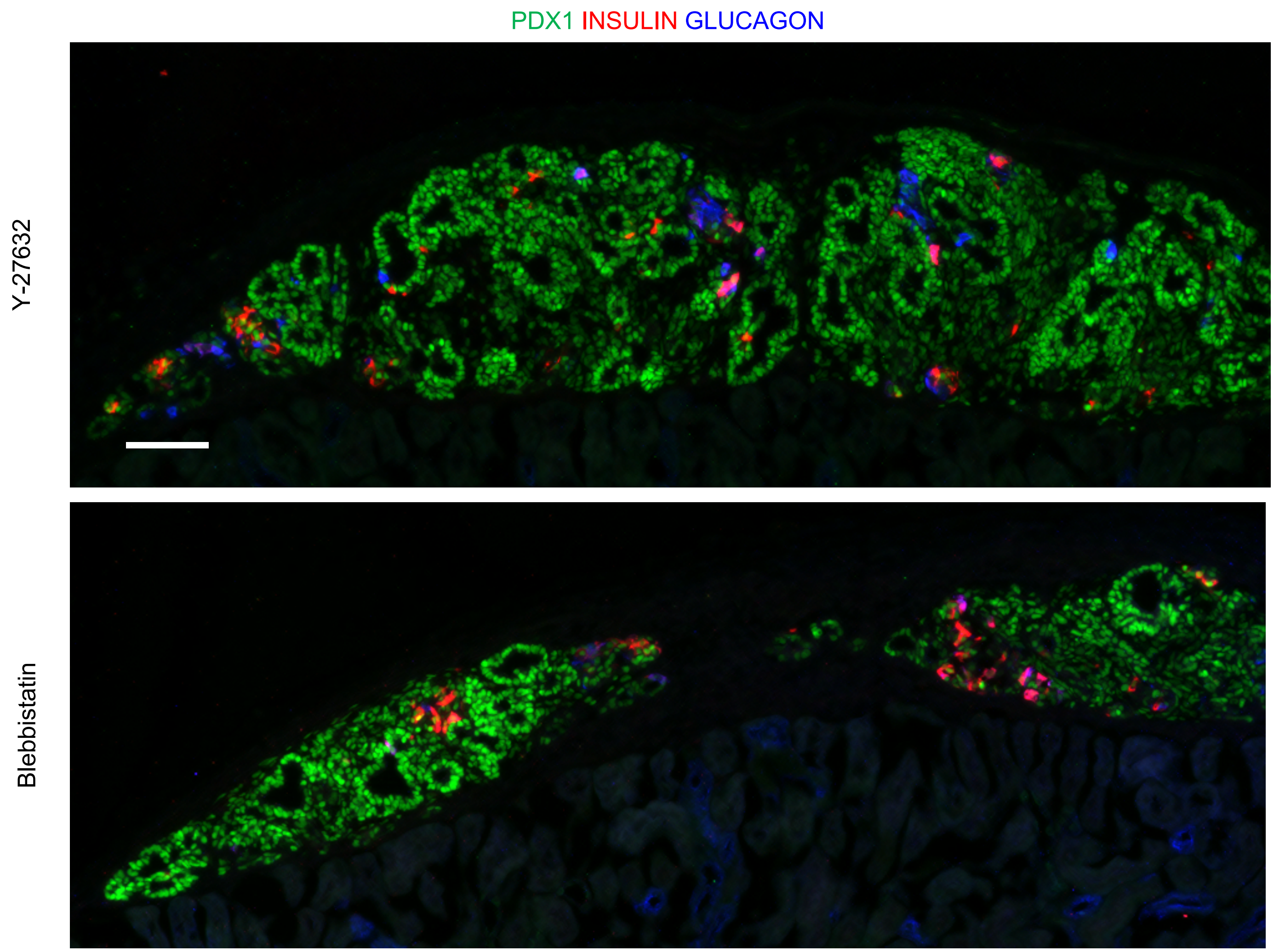
A
PDX1 INSULIN GLUCAGON
Y-27632
Blebbistatin

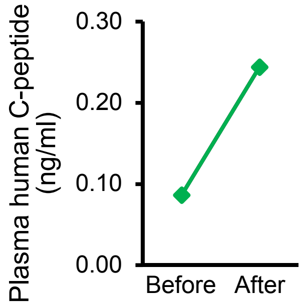
B
Plasma human C-peptide (ng/ml)
0.30
0.20
0.10
0.00
Before
After

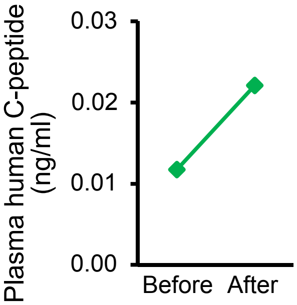
C
Plasma human C-peptide (ng/ml)
0.03
0.02
0.01
0.00
Before
After

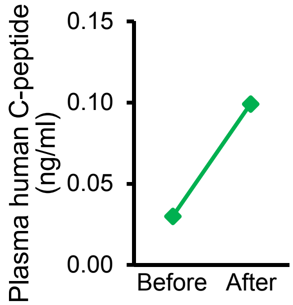
D
Plasma human C-peptide (ng/ml)
0.15
0.10
0.05
0.00
Before
After

Figure S4

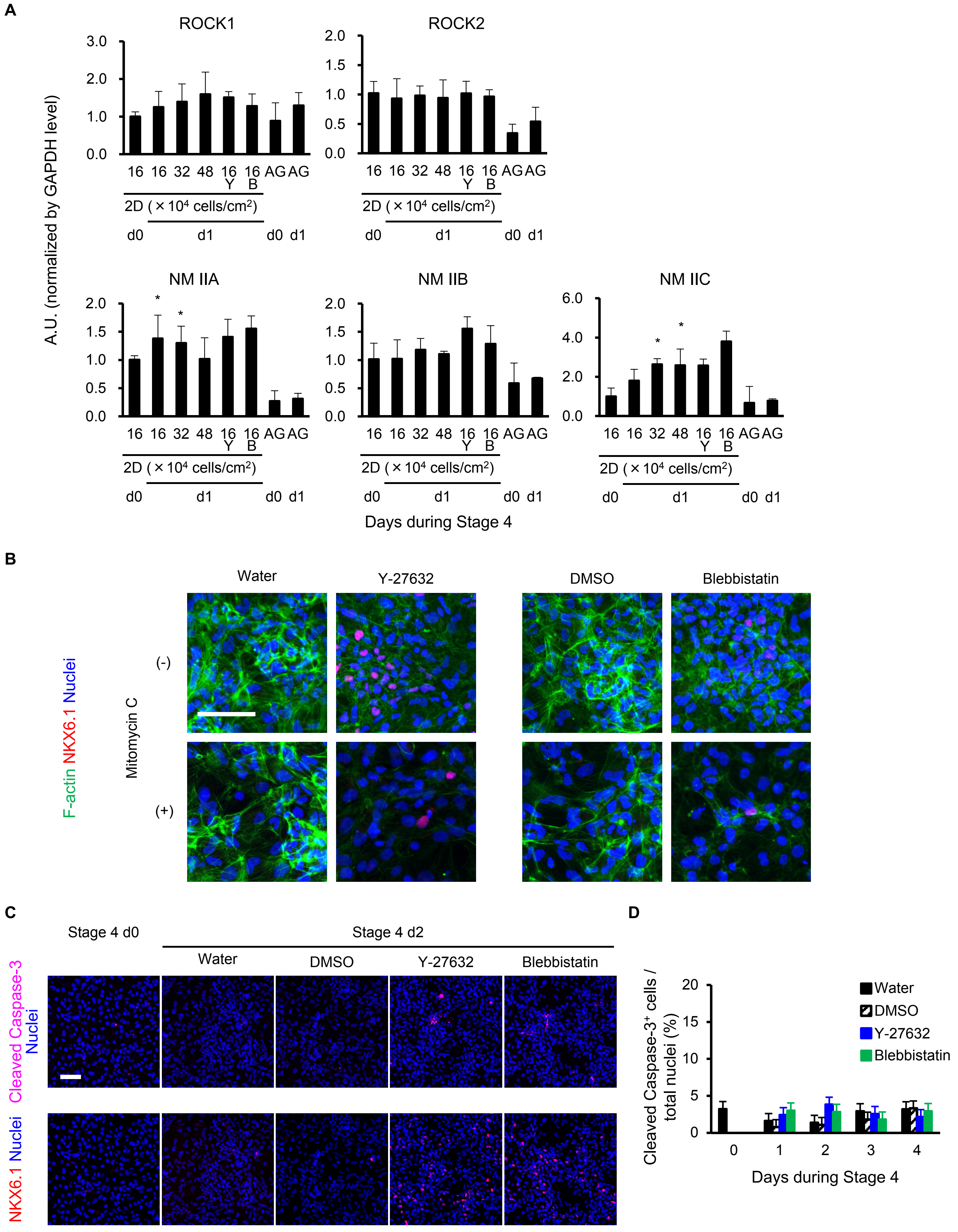
A
ROCK1
ROCK2
NM IIA
NM IIB
NM IIC
A.U. (normalized by GAPDH level)
16 16 32 48 16 16 AG AG
Y B
2D (×10⁴ cells/cm²)
d0 d1 d0 d1
Days during Stage 4
B
Water
Y-27632
DMSO
Blebbistatin
F-actin NKX6.1 Nuclei
Mitomycin C
(-)
(+)
C
Stage 4 d0
Stage 4 d2
Water
DMSO
Y-27632
Blebbistatin
Cleaved Caspase-3 Nuclei
NKX6.1 Nuclei
D
Cleaved Caspase-3⁺ cells / total nuclei (%)
Water
DMSO
Y-27632
Blebbistatin
Days during Stage 4

Supplemental Figure Legends

Figure S1 NKX6.1$^+$ cell induction does not always accompany disruption of actin fibers or cell shape changes. Related to Figure 1.

hiPSC-derived PDX1$^+$ posterior foregut cells were cultured with KGF (100 ng/ml), NOGGIN (100 ng/ml), EGF (50 ng/ml) and several cytoskeletal modulators: Latrunculin A (10 nM), Wiskostatin (1 μM), Cytochalasin D (50 μM), Blebbistatin (5 μM), BTS (50 μM), ML-7 (5 μM), W-7 (5 μM), Y-27632 (50 μM), Fasudil (50 μM), GSK269962 (10 μM), H-1152 (10 μM), Colchicine (10 nM), Nocodazole (100 nM), Docetaxel (10 μM), Paclitaxel (5 μM), Vinblastine (10 nM) and Vincristine (10 nM). The cells were fixed on Stage 4 day 4 and stained with F-actin (green), NKX6.1 (red) and nuclei (blue). Cell shapes were observed by phase contrast images. Representative images of three independent experiments. Note that NKX6.1$^+$ cells were not induced by any concentration of Latrunculin A (0.005, 0.01, 0.05, 0.1, 0.5, 1, 5 and 10 μM), Wiskostatin (0.01, 0.05, 0.1, 0.5, 1, 5, 10, 20 and 50 μM), Cytochalasin D (0.005, 0.01, 0.05, 0.1, 0.5, 1, 5, 10, 20 and 50 μM), BTS (0.01, 0.05, 0.1, 0.5, 1, 5, 10, 20, 30, 50, 80 and 100 μM), ML-7 (0.01, 0.05, 0.1, 0.5, 1, 5, 10, 20, 30 and 50 μM), W-7 (0.01, 0.05, 0.1, 0.5, 1, 5, 10, 20, 50, 80 and 100 μM), Colchicine (0.01, 0.05, 0.1, 0.5, 1, 5, 10, 20, 50, 80 and 100 μM), Nocodazole (0.01, 0.05, 0.1, 0.5, 1, 5, 10, 20 30 and 50 μM), Docetaxel (0.01, 0.05, 0.1, 0.5, 1, 5, 10, 20, 50 and 100 μM), Paclitaxel (0.01, 0.05, 0.1, 0.5, 1, 5, 10, 20, 50, 100 μM), Vinblastine (0.01, 0.05, 0.1, 0.5, 1, 5, 10, 20, 50, 80 and 100 μM) or Vincristine (0.01, 0.05, 0.1, 0.5, 1, 5, 10, 20, 50, 80 and 100 μM) (data not shown). Scale bar, 20 μm. F-actin, filamentous actin.

Figure S2 NKX6.1$^+$ cell induction does not always accompany disruption of actin fibers. Related to Figures 1 and 2.

(A) A list of main and other targets of the cytoskeletal modulators used in Figure 1. The targets are based on the Tocris Bioscience website (Bristol, United Kingdom, https://www.tocris.com/). (B) Representative images of dose dependent NKX6.1$^+$ cell induction in Y-27632-treated cells. hiPSC-derived PDX1$^+$ posterior foregut cells were cultured with KGF (100 ng/ml), NOGGIN (100 ng/ml), EGF (50 ng/ml) and Y-27632 (0, 5 and 50 μM). The cells were fixed on Stage 4 day 4 and stained with F-actin (green), NKX6.1 (red) and nuclei (blue). Low dose of Y-27632 treatment (5 μM) was effective at disrupting actin fibers but failed to induce NKX6.1$^+$ cells. Scale bar, 100 μm. (C) ROCK-NM II inhibitors effectively induced PDX1$^+$NKX6.1$^+$ cells from multiple hESC/iPSC lines. The proportion of PDX1$^+$NKX6.1$^+$ cells was quantified by flow cytometry after 4-day induction of pancreatic endoderm cells in the presence or absence of Y-27632 (50 μM) or Blebbistatin (5 μM) at Stage 4 in hESC line KhES-3 and hiPSC line Ff-I01. Abbreviations for the list in (A) are as follows. CAMKII, Ca^{2+}-calmodulin-dependent protein kinase II; MLCK, myosin light-chain kinase; N-WASP, neural Wiskott-Aldrich syndrome protein; PDE1, Ca^{2+}-calmodulin-dependent phosphodiesterase; PKA, cyclic AMP-dependent protein kinase; PKC, protein kinase C; PKG, cyclic GMP-dependent protein kinase; PRK2, Protein kinase C-related kinase 2; ROCK1, Rho-associated protein kinase 1; ROCK2, Rho-associated protein kinase 2.

Figure S3 PDX1$^+$NKX6.1$^+$ cells generated with Y-27632 or Blebbistatin develop into pancreatic epithelia *in vivo*. Related to Figure 2.

(A) Representative cryosection images of grafts 30 days after implantation were stained for the indicated markers from three individual mice for each group in one independent cohort of implantation experiments. Pancreatic endoderm cells induced with or without Y-27632 or Blebbistatin treatment on Stage 4 day 4 were dissociated and re-seeded to form cellular aggregates (3×10^4 cells/aggregate). A total of one million cells on Stage 4 day 6 were implanted under the kidney subcapsule of NOD-SCID mice. PDX1$^+$NKX6.1$^+$ cells induced with Y-27632 or Blebbistatin developed into branched pancreatic epithelia *in vivo*. Note that we could not observe the engraftment of control cells cultured at low density (1.6×10^5 cells/cm^2) without Y-27632 or Blebbistatin for four days at Stage 4 (data not shown). Scale bar, 100 μm. (B, C and D) Pancreatic endoderm cells induced with Blebbistatin treatment on Stage 4 day 4 were dissociated and re-seeded to form cellular aggregates (3×10^4 cells/aggregate). A total of three to four million cells on Stage 4 day 6 that had been pre-treated with an ALK5 inhibitor for 2 days were implanted under the kidney subcapsule of NOD-SCID mice. The plasma human C-peptide levels after 16 hours of fasting (Before) and 30 min after subsequent glucose injection (3.0 g/kg body weight, i.p.) (After) in the host mice were examined on days 120 (B), 151 (C) and 133 (D) after implantation. Data in (B)-(D) are from an individual mouse from two independent cohorts of implantation experiments.

Figure S4 Y-27632 and Blebbistatin induce NKX6.1 expression without increasing cellular proliferation or suppressing apoptosis. Related to Figures 3 and 4.

(A) Immunoblot quantification of ROCK1, ROCK2, NM IIA, NM IIB and NM IIC in Stage 4 cells. hiPSC-derived

$PDX1^+$ posterior foregut cells on Stage 3 day 2 were dissociated and re-seeded either for monolayer cultures (16–48×10^4 cells/cm^2, 2D) or to form cellular aggregates (3×10^4 cells/aggregate, AG). The cells were continuously cultured with Stage 3 treatment, except for the addition of 10 μM Y-27632. On the next day, the cells were cultured with Stage 4 with or without ROCK-NM II inhibitors. Protein abundance is normalized to the average protein abundance of re-seeded cells (16×10^4 cells/cm^2) on Stage 4 day 0. (B) Representative images of Stage 4 day 4 cells pre-treated with or without mitomycin C (47 μM, 2 h). On Stage 4 day 4, $NKX6.1^+$ cells were observed in Y-27632 (50 μM)- or Blebbistatin (5 μM)-treated cells after mitomycin C treatment, but not in their corresponding controls. (C and D) Apoptotic cells were not reduced by Y-27632 or Blebbistatin treatment. (C) Representative images of cells immunostained with an apoptotic marker, cleaved Caspase-3, from three independent experiments. (D) The percentage of cleaved Caspase-3^+ cells in (C) was analyzed by manual counting. Data are presented as the mean ± S.D. from three independent experiments (n=3) in (A) and (D). *, $P < 0.05$ versus AG on day 1. Scale bar, 100 μm. Y, Y-27632; B, Blebbistatin.

Supplemental Tables

Table S1. Antibodies used in this study.

**Antigen**	**Species**	**Source**	**Dilution**
PDX1	Goat	*AF2419, R&D Systems	1:200
NKX6.1	Mouse	*F55A12, University of Iowa	1:100
Insulin	Guinea pig	A0564, Dako	1:200
Glucagon	Mouse	G2654, Sigma-Aldrich	1:200
Nucleus	N/A	Hoechst 33342; H3570, Invitrogen	1:200
ROCK1	Rabbit	4035, Cell Signaling Technology	1:1000
ROCK2	Mouse	610624, BD Transduction Laboratories	1:2000
NM IIA	Rabbit	M8064, Sigma	1:4000
NM IIB	Rabbit	M7939, Sigma	1:4000
NM IIC	Rabbit	8189, Cell Signaling Technology	1:1000
GAPDH	Mouse	014-25524, Wako	1:5000
Cleaved Caspase-3	Rabbit	9661, Cell Signaling Technology	1:200
pMLC2 Ser^{19}	Rabbit	3671, Cell Signaling Technology	1:200

*: antibodies used for flow cytometry

Table S2. Gene names and primer sequences for qRT-PCR

**Gene name**	**Gene Symbol**	**Forward primer**	**Reverse primer**
Pancreas specific transcription factor, 1a	*PTF1A*	CCCCAGCGACCCTGATTA	GGACACAAACTCAAATGGTGG
NK6 homeobox 1	*NKX6.1*	ATTCGTTGGGGATGACAGAG	TGGGATCCAGAGGCTTATTG
Glyceraldehyde-3-phosphate dehydrogenase	*GAPDH*	GAAGGTGAAGGTCGGAGTC	GAAGATGGTGATGGGATTTC
Rho associated coiled-coil containing protein kinase 1	*ROCK1*	CTGCAACTGGAACTCAACCA	TTCTACCAATTGCGCTTGC
Rho-associated coiled-coil containing protein kinase 2	*ROCK2*	CCTGTCAAGCGTGGTAATGA	CGAATCTGGCTCTCTTCAGC
myosin, heavy chain 9, non-muscle	*MYH9*	GGGCACTGTCAAGTCCAAGT	AGCAGCACATCCTTCAGCTT
myosin, heavy chain 10, non-muscle	*MYH10*	GCAGAACAAGGAGCTGAAGG	TGCGACGGACTAATTTGTTG
myosin, heavy chain 14, non-muscle	*MYH14*	CCACAAGATGACCATTGCTG	CAGCTTTCCAGAGAGGATGC

Supplemental Experimental Procedures

hESC/iPSC culture and differentiation

The maintenance culture of a human embryonic stem cell (hESC) line, KhES-3 (Suemori et al., 2006), and a human induced pluripotent stem cell (hiPSC) line, 585A1 (Kajiwara et al., 2012), was performed as described previously (Toyoda et al., 2015). The peripheral blood-derived iPSC line Ff-I01 was generated at the Center for iPS Cell Research and Application (CiRA), Kyoto University. For on-feeder cultures, cells were grown on feeder layers of mitomycin C-treated SNL 76/7 (ECACC 07032801) in media containing Primate ES medium (ReproCELL, Yokohama, Japan) supplemented with 500 U/ml penicillin/streptomycin (P/S, Thermo Fisher Scientific, Waltham, MA) and 4 ng/ml recombinant human basic fibroblast growth factor (bFGF; Wako, Osaka, Japan). For routine passaging, hESC/iPSC colonies were dissociated by an enzymatic method with CTK dissociation solution consisting of 0.25% trypsin (Thermo Fisher Scientific), 0.1% collagenase IV (Thermo Fisher Scientific), 20% knockout serum replacement (KSR; Thermo Fisher Scientific) and 1 mM $CaCl_2$ in phosphate buffered saline (PBS) and split at a ratio between 1:6 and 1:8. For feeder-free cultures, cells were maintained with Essential 8 medium (Thermo Fisher Scientific) according to the manufacturer's instructions. For Ff-I01, cells were maintained with StemFit AK03 (Ajinomoto, Tokyo, Japan) on iMatrix-511 (Nippi, Tokyo, Japan) according to the manufacturer's instructions. Experiments with hESCs/iPSCs were approved by the ethics committee of the Department of Medicine and Graduate School of Medicine, Kyoto University. Cells were directed into key stages of pancreatic development, including definitive endoderm (Stage 1), primitive gut tube (Stage 2), posterior foregut (Stage 3) and pancreatic endoderm (Stage 4). The final protocol follows Figure 1A.

Stage 1: hESC/iPSC colonies grown on a SNL-feeder layer were first deprived of feeder cells (Suemori et al., 2006). Then, the cells were dissociated into single cells as described previously (Toyoda et al., 2015). The cells were resuspended with Stage 1 medium containing RPMI 1640 medium (NACALAI TESQUE, Kyoto, Japan) supplemented with 2% (vol/vol) B-27 Serum-Free Supplement (B27, Thermo Fisher Scientific), 50 U/ml P/S, 100 ng/ml recombinant human/mouse/rat activin A (R&D Systems, Minneapolis, MN), 3 μM CHIR99021 (Axon Medchem, Groningen, Netherlands) and 10 μM Y-27632 (Wako), seeded on BD Matrigel Basement Membrane Matrix Growth Factor Reduced (Matrigel, Becton Dickinson, Franklin Lakes, NJ)-coated plates at a density of 1×10^5 cells/cm^2 and cultured for one day. For the next two days, the cells were cultured in RPMI 1640 medium with 2% B27, 50 U/ml P/S, 100 ng/ml activin A and 1 μM CHIR99021. The day after that, the cells were cultured in RPMI 1640 medium with 2% B27, 50 U/ml P/S and 100 ng/ml activin A.

Stage 2: The cells were exposed to Improved MEM Zinc Option (iMEM) medium (Thermo Fisher Scientific) supplemented with 1% B27, 100 U/ml P/S (iMEM-B27) and 50 ng/ml keratinocyte growth factor (KGF; R&D Systems) for four days.

Stage 3: The cultures were continued for two days in iMEM-B27 with 0.5 μM 3-Keto-N-aminoethyl-N′-aminocaproyldihydrocinnamoyl cyclopamine (KAAD-CYC; Toronto Research Chemicals, Toronto, Canada), 0.5 nM 4-[(E)-2-(5,6,7,8-Tetrahydro-5,5,8,8-tetramethyl-2-naphthalenyl)-1-propenyl]-benzoic acid (TTNPB, Santa Cruz Biotechnology, Dallas, TX), 50 ng/ml KGF and 100 ng/ml NOGGIN (PeproTech, Rocky Hill, NJ). On the next day, the cells were dissociated into single cells by gentle pipetting after treatment with 0.25% trypsin–EDTA. Then, the cells were re-seeded on Matrigel-coated plates at a density of 1.6×10^5 cells/cm^2. The same inducing factors were used as described above, except for the addition of 10 μM Y-27632 to the Stage 3 treatment.

Stage 4: The cells were cultured for 1-8 days in iMEM-B27 with 100 ng/ml KGF, 100 ng/ml NOGGIN, 50 ng/ml epidermal growth factor (EGF, R&D Systems) and cytoskeletal modulators, such as 50 μM Y-27632 or 5 μM (*S*)-(-)-Blebbistatin (Blebbistatin, Toronto Research Chemicals). To evaluate various cytoskeletal modulators, the cells were subjected to four days of treatment with Latrunculin A (10 nM, Santa Cruz Biotechnology), Wiskostatin (1 μM, Santa Cruz Biotechnology), Cytochalasin D (50 μM, Santa Cruz Biotechnology), Blebbistatin (5 μM), BTS (50 μM, Santa Cruz Biotechnology), ML-7 (5 μM, Santa Cruz Biotechnology), W-7 (5 μM, Wako), Y-27632 (50 μM), Fasudil (50 μM, NACALAI TESQUE), GSK269962 (10 μM, Wako), H-1152 (10 μM, Wako), Colchicine (10 nM, Wako), Nocodazole (100 nM, Wako), Docetaxel (10 μM, Sigma), Paclitaxel (5 μM, Wako), Vinblastine (10 nM, Wako) and Vincristine (10 nM, LKT Laboratories, Paul, MN). For mitomycin C treatment, the cells were pre-treated with mitomycin C (47 μM, 2 h, Kyowa Hakko Kirin, Tokyo, Japan) before the start of Stage 4 culture.

Immunostaining

The cells were fixed with 4% paraformaldehyde (PFA) for 20 min at 4 °C. Then, immunostaining was performed as described previously (Toyoda et al., 2015). The primary antibodies used are detailed in Table S1. Nuclei were stained with Hoechst 33342 (Thermo Fisher Scientific). Filamentous-actin (F-actin) was stained with Acti-stain

phalloidin (Cytoskeletal, Denver CO). The implanted grafts were fixed with 4% PFA for 1-2 days at 4 °C. After washing with PBS, the samples were equilibrated in a 10–30% sucrose solution at room temperature for 1 h and then mounted and frozen. The frozen blocks were sectioned at 10–30 µm, and immunostaining was performed after removing the mounting medium. For quantification of the NKX6.1$^+$ cell ratios, immunostained cells were analyzed using an image analyzer CellInsight NXT (Thermo Fisher Scientific). For quantification of the cleaved Caspase-3$^+$ cell rate, immunostained cells were analyzed by manual counting.

Flow cytometry
The cells were dissociated into single cells with 0.25% trypsin–EDTA treatment, fixed, permeabilized and blocked with BD Cytofix/Cytoperm Kit (Becton Dickinson). Then, the cells were stained with the antibodies detailed in Table S1. Stained undifferentiated hiPSCs and Stage 4 day 0 cells were used as negative controls for gating.

Quantitative real-time reverse transcription polymerase chain reaction (qRT-PCR)
Total RNA was isolated from the cells with an RNeasy kit (Qiagen, Hilden, Germany), and cDNA was prepared with a ReverTra Ace qPCR RT Master Mix (TOYOBO, Osaka, Japan) and oligo (dT)20 primer according to the manufacturer's instructions. The qRT-PCR analysis was carried out with SYBR Premix Ex Taq II (Takara, Otsu, Japan). The expression of each gene was normalized to the level of *glyceraldehyde-3-phosphate dehydrogenase* (*GAPDH*) expression. The primer sequences used are shown in Table S2.

Animal studies and implantation experiments
All animal experiments were performed in accordance with the Guidelines for Animal Experiments of Kyoto University. Male 7- to 14-week-old NOD.CB17-Prkdc^{scid}/J mice (NOD–SCID, Charles River Laboratories Japan, Yokohama, Japan) were maintained on a 12-h light/dark cycle with ad libitum access to a standard irradiated diet. Mice were anesthetized with inhalable isoflurane and received implants of hiPSC-derived cell aggregates after Stage 4. The cells on Stage 4 day 4 were dissociated, and cell aggregates (3×10^4 cells/aggregate) were formed. The cell aggregates were cultured in Stage 4 medium with or without an ALK5 inhibitor (Santa Cruz Biotechnology) for an additional two days before implantation. Then, 60-200 cell aggregates per mouse were implanted under a kidney subcapsule. At 30 days after implantation, serial sections of the grafts were examined by immunostaining, as described above. The graft function was assessed by measuring human C-peptide levels in mouse plasma in response to glucose administration. The mice were fasted for >5 h, and then a 30% glucose solution was administered by intraperitoneal injection at a dose of 3.0 g/kg body weight. Blood samples were collected prior to and at 30 min after the glucose administration via a tail vein to heparinized capillaries. The plasma human C-peptide levels were analyzed by an ELISA (Mercodia, Uppsala, Sweden) according to the manufacturer's instructions. All metabolic analyses were performed in conscious and restrained animals.

Immunoblots
The cells were processed with a rubber scraper in RIPA Buffer (Wako) with Protease Inhibitor Cocktail Set III (Wako) and Phosphatase Inhibitor Cocktail Solution I (Wako), and then subjected to Bioruptor UCD-250HSA (Tosyodenki, Kanagawa, Japan) on ice. After centrifugation (15,000 × *g*) at 4 °C for 15 min, the supernatant was collected. Lysate protein concentrations were determined by the Bradford method (Bradford, 1976) using the dye reagent XL-Bradford (KY-1040, APRO SCIENCE, Naruto, Japan). Lysates (2-4 g of protein) were separated by SDS-PAGE before immunoblotting (Laemmli, 1970). Antibody-bound proteins were visualized with chemiluminescence detection reagents (GE Healthcare, Buckinghamshire, U.K.) and detected using the LAS-4000 mini (Fujifilm, Tokyo, Japan). Images were quantitated by densitometry (Multi Gauge Ver. 3.2, Fujifilm). Protein expressions were quantified relative to the loading control and normalized by GAPDH levels. Fold increases are expressed relative to the average of the lysates from monolayer culture (1.6×10^5 cells/cm^2) on Stage 4 day 0. The primary antibodies used in this study are detailed in Table S1.

Statistics
Data are expressed as the means ± standard deviation or standard error. Statistical analyses were performed using ratio paired t test, one-way analysis of variance (ANOVA), two-way ANOVA or two-way repeated measure ANOVA (Prism7, GraphPad Software, San Diego). When differences between means were detected by one- or two-way analysis of variances, Dunnett's or Tukey's multiple comparison test as appropriate was used for post hoc testing. Differences between groups were considered significant when $p < 0.05$.

Supplemental References

Bradford, M.M. (1976). A rapid and sensitive method for the quantitation of microgram quantities of protein utilizing the principle of protein-dye binding. Anal Biochem *72*, 248-254.

Kajiwara, M., Aoi, T., Okita, K., Takahashi, R., Inoue, H., Takayama, N., Endo, H., Eto, K., Toguchida, J., Uemoto, S., *et al.* (2012). Donor-dependent variations in hepatic differentiation from human-induced pluripotent stem cells. Proc Natl Acad Sci U S A *109*, 12538-12543.

Laemmli, U.K. (1970). Cleavage of structural proteins during the assembly of the head of bacteriophage T4. Nature *227*, 680-685.

Suemori, H., Yasuchika, K., Hasegawa, K., Fujioka, T., Tsuneyoshi, N., and Nakatsuji, N. (2006). Efficient establishment of human embryonic stem cell lines and long-term maintenance with stable karyotype by enzymatic bulk passage. Biochem Biophys Res Commun *345*, 926-932.
