# Supplementary material for: Rho-Associated Kinases and Non-muscle Myosin IIs Inhibit the Differentiation of Human iPSCs to Pancreatic Endoderm
Source: Stem Cell Reports. 2017 Aug 8;9(2):419–28. doi: 10.1016/j.stemcr.2017.07.005 (PMC5550204; doi:10.1016/j.stemcr.2017.07.005)
Supplement: Document S2. Article plus Supplemental Information [file mmc2.pdf]

Please cite this article in press as: Toyoda et al., Rho-Associated Kinases and Non-muscle Myosin IIs Inhibit the Differentiation of Human iPSCs to Pancreatic Endoderm, Stem Cell Reports (2017), http://dx.doi.org/10.1016/j.stemcr.2017.07.005

Stem Cell Reports

ISSCR

Report

OPEN ACCESS

Rho-Associated Kinases and Non-muscle Myosin IIs Inhibit the Differentiation of Human iPSCs to Pancreatic Endoderm

Taro Toyoda,[1,*] Azuma Kimura,[1] Hiromi Tanaka,[1] Tomonaga Ameku,[1] Atsushi Mima,[1] Yurie Hirose,[1] Masahiro Nakamura,[1] Akira Watanabe,[1] and Kenji Osafune[1,*]

[1]Center for iPS Cell Research and Application (CiRA), Kyoto University, 53 Kawahara-cho, Shogoin, Sakyo-ku, Kyoto 606-8507, Japan

*Correspondence: t.toyoda@cira.kyoto-u.ac.jp (T.T.), osafu@cira.kyoto-u.ac.jp (K.O.)

http://dx.doi.org/10.1016/j.stemcr.2017.07.005

SUMMARY

There has been increasing success with the generation of pancreatic cells from human induced pluripotent stem cells (hiPSCs); however, the molecular mechanisms of the differentiation remain elusive. The purpose of this study was to reveal novel molecular mechanisms for differentiation to PDX1$^+$NKX6.1$^+$ pancreatic endoderm cells, which are pancreatic committed progenitor cells. PDX1$^+$ posterior foregut cells differentiated from hiPSCs failed to differentiate into pancreatic endoderm cells at low cell density, but Rho-associated kinase (ROCK) or non-muscle myosin II (NM II) inhibitors rescued the differentiation potential. Consistently, the expression of phosphorylated myosin light chain 2 and NM IIA was downregulated in aggregation culture. Notably, the soluble factors we tested were substantially effective only with ROCK-NM II inhibition. The PDX1$^+$NKX6.1$^+$ cells induced with NM II inhibitors were successfully engrafted and maturated *in vivo*. Taken together, these results suggest that NM IIs play inhibitory roles for the differentiation of hiPSCs to pancreatic endoderm cells.

INTRODUCTION

Pancreatic cells generated from pluripotent stem cells, such as human embryonic stem cells (hESCs) and induced pluripotent stem cells (hiPSCs), are considered a promising cell source for regenerative therapies. Recent advances in regenerative medicine research have demonstrated that guided differentiation can recapitulate normal developmental stages and enables the generation of pancreatic cells, including mature type pancreatic beta cells *in vitro* (Pagliuca et al., 2014; Rezania et al., 2014). Among these stages, the cell type in pancreatic bud formation is crucial, since these cells are the earliest stage of pancreatic endoderm cells and considered committed to differentiate into only pancreatic lineages (Kelly et al., 2011; Rezania et al., 2013). Several reports have shown the efficient induction of PDX1$^+$NKX6.1$^+$ pancreatic endoderm cells, which correspond to cells at the stages from pancreatic bud to branched epithelia, from hESCs/iPSCs (Nostro et al., 2015; Pagliuca et al., 2014; Rezania et al., 2014; Russ et al., 2015; Toyoda et al., 2015). However, the molecular mechanisms regulating this differentiation remain elusive, which potentially causes unstable manipulation of the cells and contamination of other cell types, thus hampering basic research and clinical application.

The cellular morphology and physical microenvironment dramatically change during differentiation. In pancreas development, the first step of organogenesis is the formation of the pancreatic bud (Villasenor et al., 2010). A pre-pancreatic region at gut tube endoderm composes a single layer of epithelial cells that express *PDX1*. With the progress in pancreatic specification, the epithelium rapidly thickens to form an aggregation of cells called the pancreatic bud, which concomitantly express the pancreas-specific markers *NKX6.1* and *PTF1A*. This process is closely linked to changes in cell shape from cuboidal to columnar and squamous (Villasenor et al., 2010) and changes in the microenvironment, such as cell-to-cell interactions, matrix stiffness, and cell polarity. It is possible that these physical changes lead to modifications in intracellular signaling, resulting in modulation of the propensity for differentiation into pancreatic lineages (Cortijo et al., 2012; Kesavan et al., 2009). Supporting this idea, we previously showed that high cell density or aggregation promotes the differentiation of PDX1$^+$ posterior foregut cells to the earliest stage of PDX1$^+$NKX6.1$^+$ pancreatic endoderm cells in hESC/iPSC differentiation cultures (Toyoda et al., 2015). In either high-cell-density or aggregation culture, cells are condensed, suggesting a different physical environment from that in low-cell-density cultures. In many cell types, it is suggested that manipulation of the cellular morphology or physical microenvironment affects the cell state and differentiation propensity via cytoskeletal changes (Connelly et al., 2010; Maharam et al., 2015; Sun et al., 2014). More specifically, genetic and chemical modifications of cytoskeleton regulators disrupt progenitor cell proliferation, organ size, and composition of the differentiated cells in the developing pancreas (Kesavan et al., 2009; Petzold et al., 2013; Shih et al., 2016). Thus, proper cytoskeletal regulation is required for normal pancreatic organogenesis; however, the roles of the cytoskeleton in the formation of pancreatic endoderm remain unclear.

In the current study, we tested the hypothesis that molecules related to the cytoskeleton regulate the differentiation

This is an open access article under the CC BY license (http://creativecommons.org/licenses/by/4.0/).

STEMCR 822

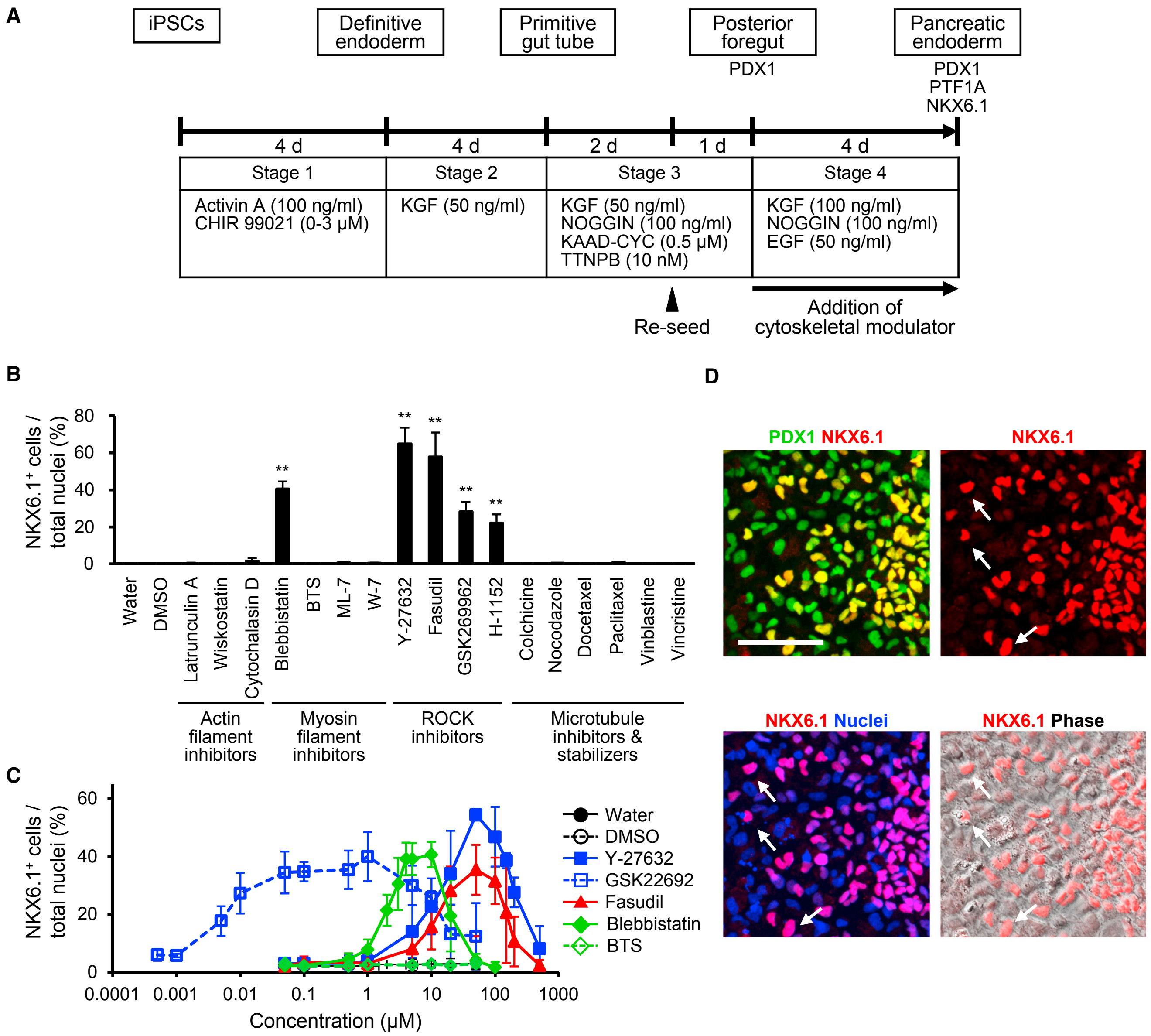

Figure 1. ROCK Signaling Inhibitors Promote PDX1$^+$NKX6.1$^+$ Cell Induction from PDX1$^+$ Posterior Foregut Cells

(A) A schematic diagram of the procedure used to differentiate pancreatic endoderm cells from hiPSCs.

(B and C) Quantification of the proportion of NKX6.1$^+$ cells by an image analyzer. Note that water, DMSO, and BTS treatment did not induce NKX6.1$^+$ cells at any concentration in (C).

(D) PDX1$^+$NKX6.1$^+$ cells were localized in sparse areas (arrows) in Y-27632-treated cells in monolayer culture.

Data are presented as the mean ± SD from three independent experiments in (B) and (C). **$p < 0.01$ versus water. Scale bar, 100 μm. See also Figures S1 and S2.

of hESC/iPSC-derived PDX1$^+$ posterior foregut cells to PDX1$^+$NKX6.1$^+$ pancreatic endoderm cells. We found that the expression of non-muscle myosin IIs (NM IIs) is downregulated in aggregation cultures and that chemical inhibition of Rho-associated kinases (ROCKs) and NM IIs promotes differentiation to pancreatic endoderm cells, mimicking the effect of aggregation cultures.

RESULTS

ROCK-NM II Inhibitors Facilitate the Differentiation of Posterior Foregut to Pancreatic Endoderm Cells

PDX1$^+$ posterior foregut cells re-seeded at low cell densities (1.6×10^5 cells/cm^2) were treated with three soluble factors: KGF, NOGGIN, and EGF (Figure 1A). As expected,

STEMCR 822

this cell density was too low to induce NKX6.1+ cells even after 4 days of treatment (water- or DMSO-treated group in Figure 1B) (Toyoda et al., 2015). To investigate whether modulation of the cytoskeleton promotes differentiation to pancreatic endoderm cells, we treated the PDX1+ cells with inhibitors of actin and myosin II filaments and ROCK and with microtubule inhibitors/stabilizers in addition to the aforementioned three factors for 3 days. We found one myosin II inhibitor (Blebbistatin) and four ROCK inhibitors (Y-27632, Fasudil, GSK269962, and H-1152) increased the proportion of NKX6.1+ cells, while a muscle-type myosin II inhibitor, BTS, and a low dose of Y-27632, which induced actin disassembly, failed to induce NKX6.1+ cells (Figures 1B, S1, S2A, and S2B). These results suggest that the signaling of ROCKs and downstream NM IIs regulates differentiation into NKX6.1+ cells, although we cannot exclude the possibility that the inhibitors used in this study may target molecules other than ROCK-NM II (Figure S2A). GSK269962, which has the highest affinity for ROCK (half-maximal inhibitory concentration [IC_{50}] for ROCK1 of GSK269962, Y-27632, and Fasudil: 1.6, 150, and 300 nM, respectively), had the most potent inducing activity (Figure 1C) (Doe et al., 2007; Jacobs et al., 2006), supporting the involvement of ROCK-NM II signaling. Cells treated with ROCK-NM II inhibitors tended to be small, but not all small cells were NKX6.1+ and large NKX6.1+ cells were also observed (Figures 1D and S1 and data not shown). Notably, NKX6.1+ cells were observed in a relatively sparse area as well as a high-density area (Figure 1D), suggesting that cell aggregation is not necessary for NKX6.1+ cell induction with treatment of ROCK-NM II inhibitors.

NKX6.1+ Cells Induced with ROCK-NM II Inhibitors Show Developmental Potential Consistent with Pancreatic Endoderm Cells

Next, we validated the developmental potential of NKX6.1+ cells induced with ROCK-NM II inhibitors. The mRNA expression of *PTF1A*, a pancreatic endoderm cell marker, was associated with an increase in the proportion of NKX6.1+ cells in ROCK-NM II inhibitor-treated cells, whereas the expression of a ductal multipotent progenitor marker, *SOX9*, was increased in all groups with time (Figures 2A and 2B). Flow cytometric analysis demonstrated that all NKX6.1+ cells induced by ROCK-NM II inhibitors co-expressed PDX1 (Figures 2C and 2D). The positive effects of ROCK-NM II inhibition on differentiation into NKX6.1+ cells were reproduced in hESC line KhES-3 and hiPSC line Ff-I01 (Figure S2C). Moreover, the cells treated with Y-27632 or Blebbistatin at stage 4 were able to form PDX1+ tubular structures that possess INSULIN+ and GLUCAGON+ endocrine cells, which is reminiscent of human embryonic pancreatic epithelia, 30 days after implantation into the renal subcapsule of immunodeficient mice (Figures 2E and S3A). Plasma human C-peptide levels, which indicate human insulin secretion, gradually increased over time and eventually responded to changes in blood glucose levels in mice implanted with Blebbistatin-treated cells (Figures 2F, 2G, and S3B–S3D). These results suggest that the NKX6.1+ cells induced with ROCK-NM II inhibitors are pancreatic endoderm cells that have the developmental potential to differentiate into pancreatic epithelia and mature into β cells *in vivo*.

NM II Signaling Molecules Are Downregulated in Aggregation Culture

ROCK-NM II inhibition facilitates differentiation into pancreatic endoderm cells without high-cell-density or aggregation cultures. To examine whether ROCK-NM II signaling is inhibited in high-cell-density or aggregation cultures, we analyzed the mRNA expression of genes encoding ROCK1 and ROCK2 (*ROCK1* and *ROCK2*) and NM IIA, NM IIB, and NM IIC (*MYH9*, *MYH10*, and *MYH14*) in monolayer-cultured cells at different cell densities (8–48 × 10^4 cells/cm^2) and in cellular aggregates on stage 4 day 0. We found a tendency for the mRNA expression of *MYH9* and *MYH14* to decrease as the cell density increased (Figure 3A). Notably, the mRNA expression of *MYH9* and *MYH14* was lowest in the cellular aggregates. Interestingly, the mRNA expression of all five genes was significantly lower in the cellular aggregates than in low-cell-density monolayer cultures at stage 4 (Figure 3B). Consistent with these findings, the protein levels of NM IIA and NM IIC, as evaluated by western blotting, were lowest in the cellular aggregates (Figures 3C and S4A), and the levels of phosphorylated myosin light chain 2 (pMLC2), which indicates ROCK activity (Amano et al., 1996), and NM IIA, as evaluated by immunostaining, were weaker in high-cell-density and aggregation cultures than in low-cell-density cultures (Figure 3D). The difference in the results of NM IIA expression with high-cell-density cultures between western blotting and immunostaining is possibly due to the different sensitivity and targets of each method. Western blotting evenly detects all cellular NM IIA molecules, whereas immunostaining emphasizes accumulated NM IIA molecules such as polymeric fibers compared with monomers. Taken together, these results suggest that signaling related to ROCK-NM II is suppressed multiple ways by aggregation cultures.

Differentiation Mechanisms by which ROCK-NM II Inhibitors Induce Pancreatic Endoderm Cells Mimic Aggregation Effects

We previously found that the signals induced by cell aggregation cultures for pancreatic endoderm cell induction are different from those induced by soluble factors (KGF,

STEMCR 822

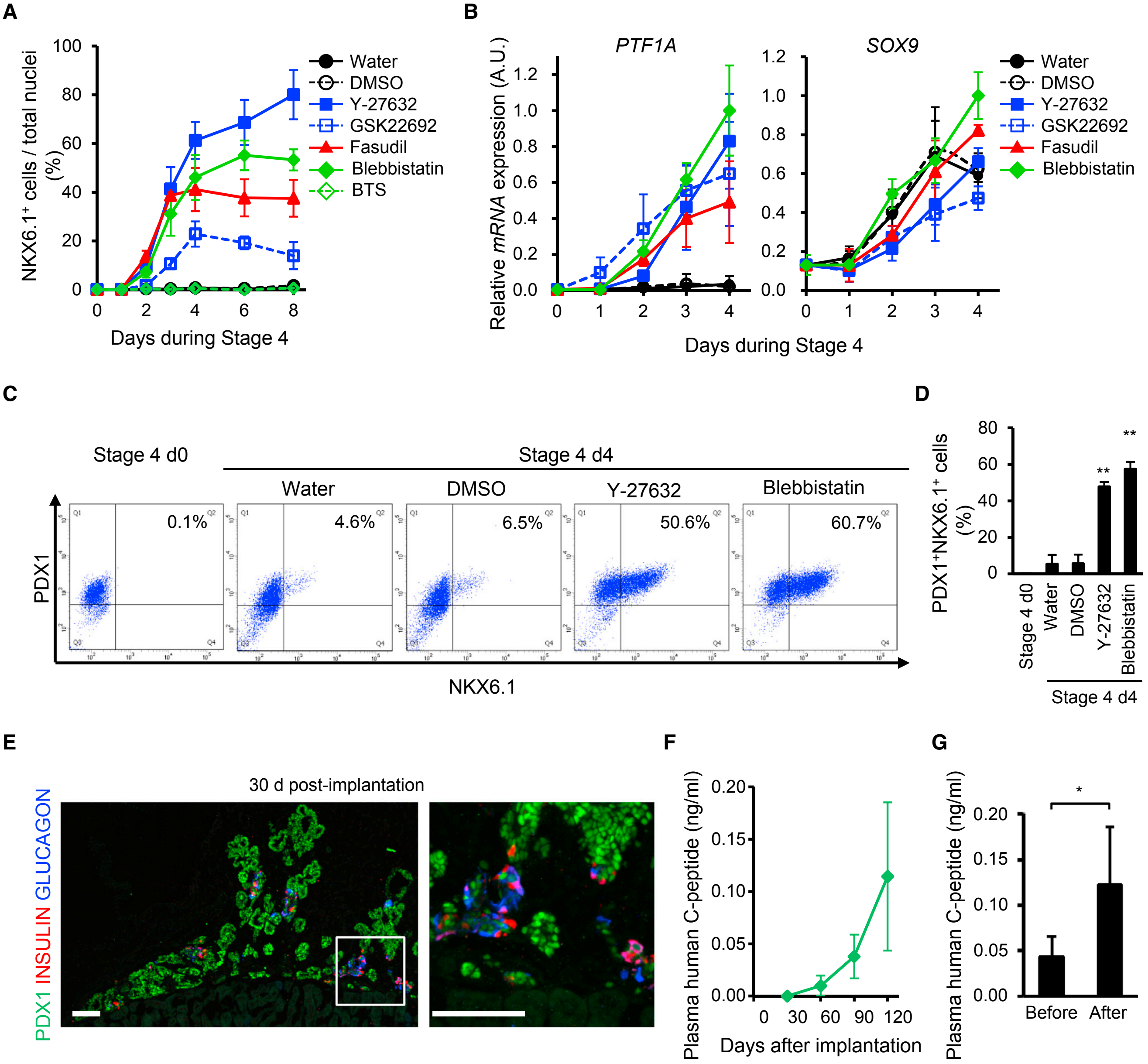

Figure 2. NKX6.1⁺ Cells Induced with ROCK-NM II Inhibitors Show Developmental Potential Consistent with Pancreatic Endoderm Cells

(A) Time course for NKX6.1^{+} cell induction by ROCK-NM II inhibitors was analyzed by an image analyzer. Note that water, DMSO, and BTS treatment did not induce NKX6.1^{+} cells at any concentration.

(B) The mRNA expression of a pancreatic endoderm marker, *PTF1A*, and a ductal progenitor marker, *SOX9*.

(C and D) The proportion of PDX1^{+}NKX6.1^{+} cells was analyzed by flow cytometry. Representative dot plots (C) and quantification of the induction efficiency (D).

(E) PDX1^{+}NKX6.1^{+} cells induced with Blebbistatin developed into branched pancreatic epithelia *in vivo*. A representative cryosection image of grafts 30 days after implantation from three independent experiments was stained for the indicated markers. The right panel shows a magnified image of the boxed area in the left panel.

(F and G) Plasma human C-peptide levels in host mice at various time points after implantation (F) and before and after glucose injection on days 120–151 after implantation (G).

Data are presented as the mean ± SD from three independent experiments in (A, B, and D) and as the mean ± SE of three mice from two independent cohorts of implantation experiments in (F) and (G). *$p < 0.05$. **$p < 0.01$ versus water. Scale bars, 100 μm. See also Figures S2 and S3.

STEMCR 822

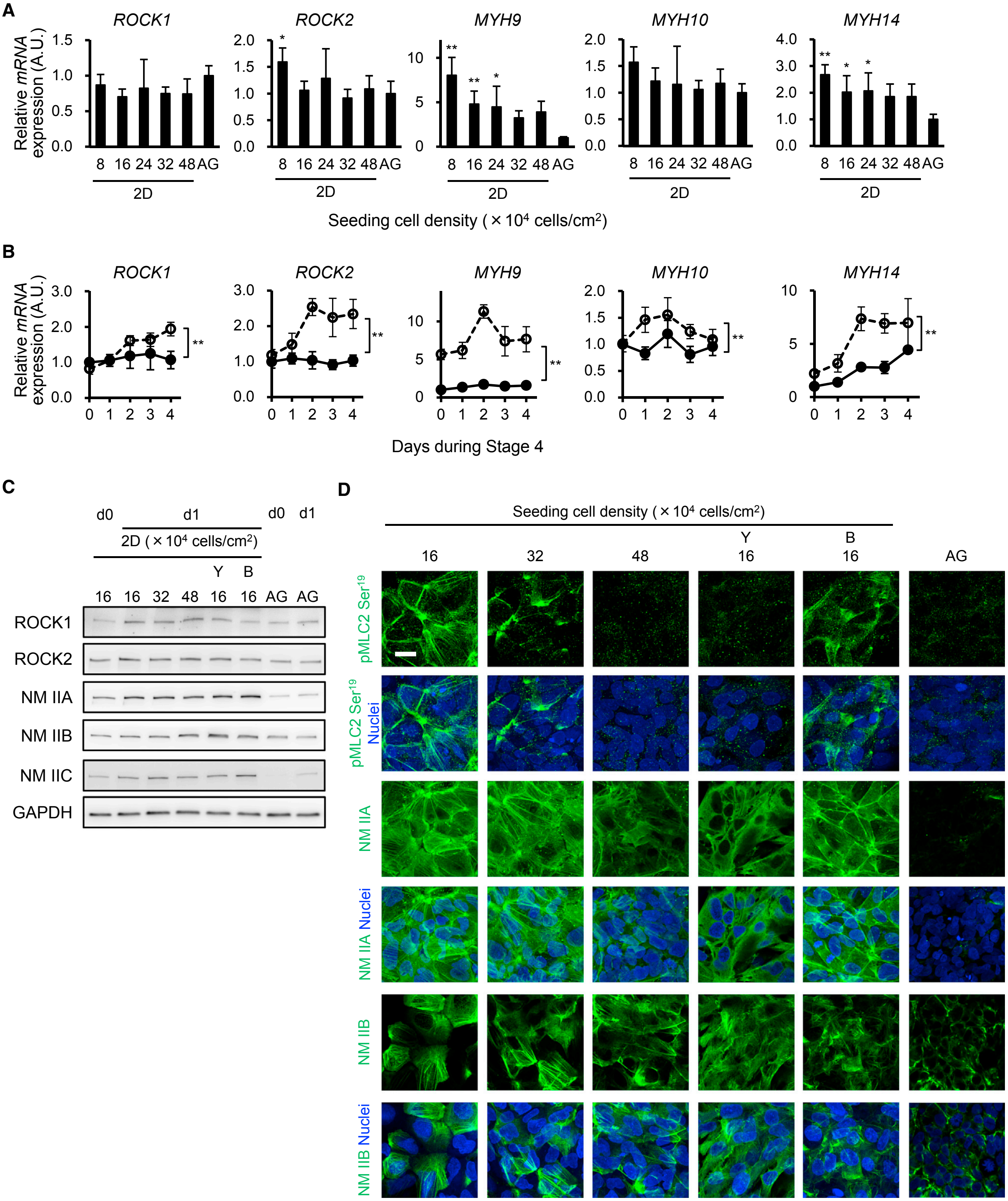

Figure 3. ROCK-NM II Signaling Is Downregulated in Aggregation Cultures

(A and B) PDX1⁺ posterior foregut cells were re-seeded either for monolayer cultures (2D) or to form cellular aggregates (3×10^4 cells/aggregate, AG). The next day, the cells were exposed to stage 4 treatment without ROCK-NM II inhibitors. The mRNA expression of genes

(legend continued on next page)

NOGGIN, and EGF) (Toyoda et al., 2015). The combination of cell aggregation cultures with any one of these soluble factors upregulated *NKX6.1* expression. Similar to the effects of cell aggregation, a combination of ROCK-NM II inhibitors and one soluble factor also increased the expression of *NKX6.1* (Figure 4A). These results suggest that the signals regulated by ROCK-NM II inhibition are independent of those induced by the three aforementioned factors.

Next, we examined whether ROCK-NM II inhibition works through high-cell-density or aggregation effects caused by cell proliferation. The expression of *NKX6.1* was increased by Y-27632 or Blebbistatin treatment under the inhibition of cell proliferation by pre-treatment with a mitotic inactivator, mitomycin C (Figures 4B–4D and S4B). Y-27632 or Blebbistatin treatment did not prevent apoptosis, as evaluated by immunostaining against an apoptotic marker, cleaved caspase-3 (Figures S4C and S4D). In addition, we analyzed the relationship between the proportion of $PDX1^{+}NKX6.1^{+}$ cells and cell density on stage 4 day 4. Scatterplots demonstrated that the efficiency of $PDX1^{+}NKX6.1^{+}$ cell induction was higher in ROCK-NM II inhibitor-treated cells than the corresponding controls (water- or DMSO-treated cells) at similar cell densities (Figure 4E). These findings support the idea that ROCK-NM II inhibition induces pancreatic endoderm cells by mechanisms that mimic aggregation effects but not by increasing the cell number, resulting in high-cell-density or aggregation effects.

DISCUSSION

We previously reported that high-cell-density or aggregation culture is beneficial for the induction of $PDX1^{+}$ posterior foregut cells to $PDX1^{+}NKX6.1^{+}$ pancreatic endoderm cells, however, the molecular mechanisms were undetermined (Toyoda et al., 2015). In the present study, we found that ROCK-NM II inhibition facilitated $PDX1^{+}NKX6.1^{+}$ cell induction even in low-cell-density cultures. In addition, ROCK-NM II inhibition effectively induced differentiation when combined with known soluble factors (KGF, EGF, and NOGGIN), which indicates a similar mechanism to that of aggregation cultures. These results suggest that ROCK-NM II inhibition is an independent factor for pancreatic endoderm cell induction, leading us to propose that ROCK-NM II inhibition may be one of the mechanisms underlying the effective pancreatic endoderm cell induction seen in aggregation cultures.

Two models can explain the mechanisms with which ROCK-NM II inhibition induces pancreatic endoderm cells. In one model, ROCK-NM II inhibition increases the cell number, resulting in the high-cell-density or aggregation condition that is favorable for differentiation. In the other model, ROCK-NM II inhibition directly regulates differentiation signals. *NKX6.1* mRNA expression was induced by Y-27632 or Blebbistatin under the inhibition of cell proliferation or without the disruption of apoptosis, suggesting that *NKX6.1* induction is at least in part via a mechanism independent of cell density. Consistently, ROCK-NM II inhibition had a higher induction efficiency of $PDX1^{+}NKX6.1^{+}$ cells than corresponding controls with the same cell densities, including cell densities at lower ranges. Based on these observations, it is likely that ROCK-NM II inhibition directly regulates signals mimicking those induced by aggregation cultures.

The priming of cell differentiation and commitment into certain cell types are coordinated by the balance of inducing and inhibiting signals. We found that cells in high-cell-density or aggregation cultures, which favor differentiation to pancreatic endoderm cells, had lower ROCK activity than cells in low-cell-density cultures, and the expression of NM IIA mRNA and protein was lowest in aggregation cultures. Therefore, it is reasonable to assume that NM IIA suppresses differentiation into pancreatic endoderm cells. We also found that low-cell-density cultures increased ROCK-NM II mRNA expression with time. Based on these findings, we propose that binding to substances other than the surface of surrounding cells may induce cytoskeletal modifications, including the upregulation of ROCK-NM II, which in turn suppresses NKX6.1 expression. Three-dimensional cultures are often used to mimic the *in vivo* microenvironment, and the benefits of such cultures have been reported in other cell types (Ogawa et al., 2013; Schyschka et al., 2013). Our findings shed light on the molecular mechanisms that give three-dimensional cultures advantages over two-dimensional cultures.

Although the detailed mechanisms by which NM IIA suppresses differentiation into pancreatic endoderm cells remain to be elucidated, we assume that NM II-associated proteins or downstream molecules may act as transcriptional regulators that repress *NKX6.1* gene expression either directly or indirectly. Some reports have described

encoding ROCKs and NM IIs in the cells on stage 4 day 0 (A) and its time course in AG (black circle, solid line) and 2D (1.6×10^5 cells/cm^2, white circle, dotted line) (B).
(C and D) Representative images of the expression levels of ROCK and NM II proteins on stage 4 days 0 and 1 (C) and ROCK downstream molecules on stage 4 day 1 (D) of three independent experiments.
Data are presented as the mean ± SD from four independent experiments in (A) and (B). *$p < 0.05$, **$p < 0.01$ versus AG. Y, Y-27632 (50 μM). B, Blebbistatin (5 μM). Scale bar, 20 μm. See also Figure S4.

Please cite this article in press as: Toyoda et al., Rho-Associated Kinases and Non-muscle Myosin IIs Inhibit the Differentiation of Human iPSCs to Pancreatic Endoderm, Stem Cell Reports (2017), http://dx.doi.org/10.1016/j.stemcr.2017.07.005

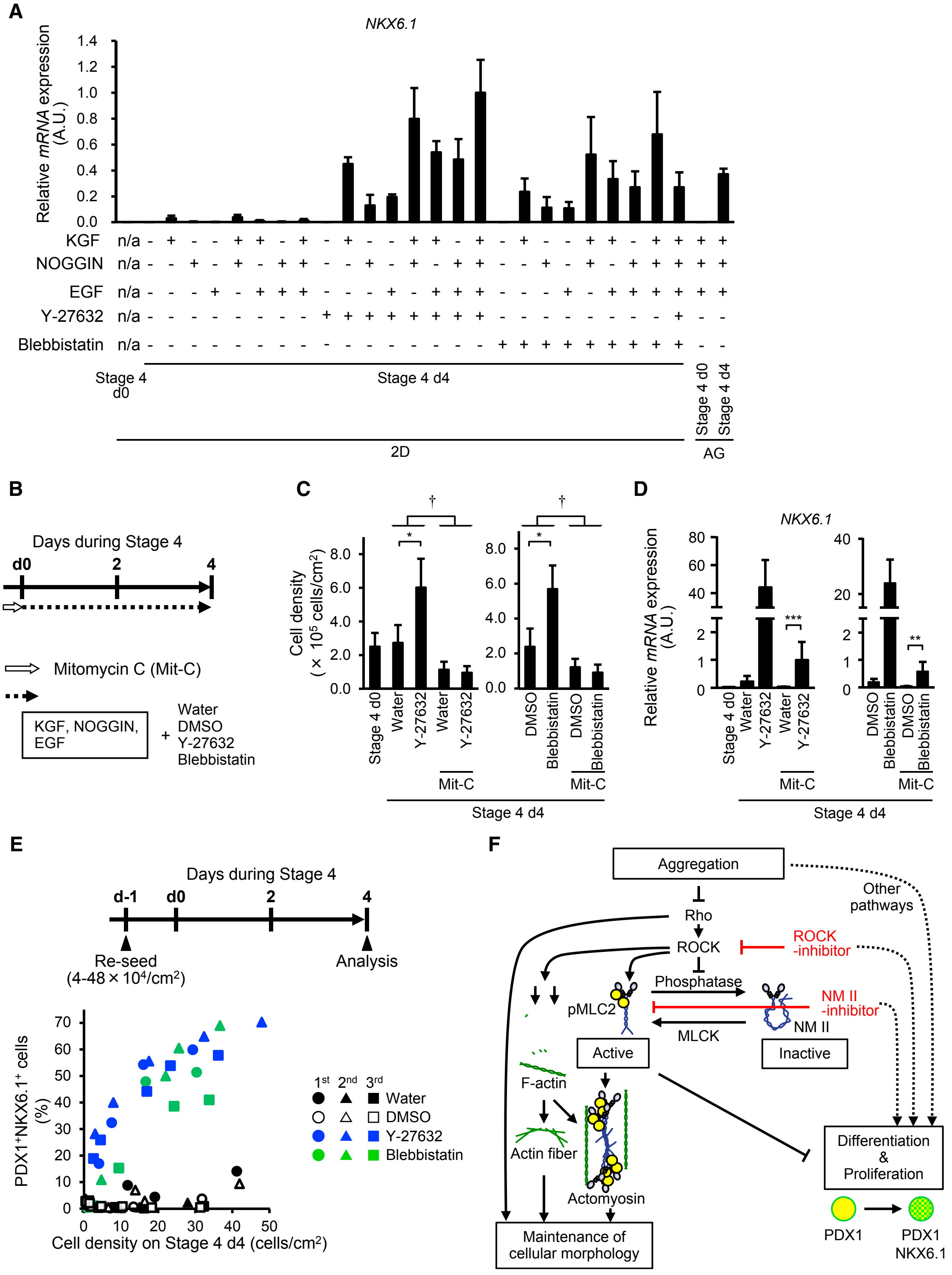

Figure 4. ROCK-NM II Inhibitors Induce NKX6.1⁺ Cells via Proliferation-Independent Mechanisms

(A) mRNA expression of *NKX6.1* in cells treated with various combinations of soluble factors (100 ng/mL KGF, 100 ng/mL NOGGIN, and 50 ng/mL EGF) and ROCK-NM II inhibitors (50 μM Y-27632 and 5 μM Blebbistatin) for 4 days of stage 4 monolayer culture.

(B) A schematic diagram of the procedures for (C) and (D). PDX1^{+} posterior foregut cells were pre-treated with mitomycin C (47 μM, 2 hr) to inhibit proliferation before pancreatic endoderm cell induction.

(legend continued on next page)

the detailed molecular mechanisms by which alterations in cytoskeletal structures regulate cellular differentiation. In mouse dedifferentiated fat cells, the disruption of actin fibers drives adipogenic differentiation through an increase in interactions between monomeric G-actin and megakaryoblastic leukemia 1 protein (Nobusue et al., 2014). In mesenchymal stem cells, information on the cell shape and soft extracellular matrix environment is transduced by two transcriptional factors, Yes-associated protein (YAP) and transcriptional coactivator with PDZ-binding motif (TAZ), which enables adipogenic differentiation (Dupont et al., 2011). Although G-actin binding protein and the YAP/TAZ system are attractive candidate mediators, we found that PDX1^{+}NKX6.1^{+} cell induction is specifically regulated by the inhibition of ROCKs and NM IIs, and not by any actin fiber or microtubule disassembling reagents. In addition, we could not find any association between cell shape or size with NKX6.1^{+} cell induction when cells were treated with cytoskeletal inhibitors. Therefore, the involvement of G-actin binding protein or the YAP/TAZ system is unlikely. A previous study reported that Fyn kinase downregulates ROCK-NM II during oligodendrocyte morphological differentiation in mice (Wang et al., 2012). Another report demonstrated that an NM II isoform, MYH9, interacts with nuclear protein Thy28/Thyn1, and that this complex binds to *Pax5* 1A promoter to suppress *Pax5* expression in chicken B cells (Fujita et al., 2015). Similarly, the elucidation of regulatory molecules or binding partners for NM II may provide new transcriptional regulating mechanisms of pancreatic development.

The efficiency of NKX6.1^{+} cell induction tended to be high at high cell density, even in ROCK-NM II inhibitor-treated cells, suggesting ROCK-NM II independent effects on cell density. One possible explanation for these effects is that NKX6.1^{+} cells may have high proliferation capacity at high cell density. Another explanation considers high-cell-density signals through both ROCK-NM II and other signaling pathways.

In summary, treatment with ROCK-NM II inhibitors or aggregation cultures that downregulate ROCK-NM II signaling facilitates the differentiation of hiPSC-derived PDX1^{+} posterior foregut cells to PDX1^{+}NKX6.1^{+} pancreatic endoderm cells. We propose that insufficient cell density may induce NM II activation, suppressing differentiation to pancreatic endoderm cells (Figure 4F). In this study, we demonstrated that ROCK-NM II signals associated with physical contexts regulate the differentiation of hESCs/iPSCs into pancreatic linages. Our findings should contribute to the development of stable cell supplies for hESC/iPSC-derived pancreatic cells.

EXPERIMENTAL PROCEDURES

hESC/iPSC Culture and Differentiation

An hESC line KhES-3 and two hiPSC lines 585A1 and Ff-I01 were maintained as described previously (Toyoda et al., 2015). Experiments with hESCs/iPSCs were approved by the ethics committee of the Department of Medicine and Graduate School of Medicine, Kyoto University. Cells were directed into key stages of pancreatic development as described previously with modifications (Figure 1A; Supplemental Experimental Procedures).

Immunostaining

Cells were fixed with 4% paraformaldehyde (PFA) for 20 min at 4°C. The implanted grafts were fixed with 4% PFA for 1–2 days at 4°C, then the samples were equilibrated in a 10%–30% sucrose solution, mounted, and frozen. The frozen blocks were sectioned at 10 μm. Immunostaining was performed as described previously (Toyoda et al., 2015). The primary antibodies used are detailed in Table S1. Filamentous-actin (F-actin) was stained with Acti-stain phalloidin (Cytoskeletal, Denver, CO).

Flow Cytometry

Cells were dissociated into single cells with 0.25% trypsin–EDTA treatment, fixed, permeabilized and blocked with a BD Cytofix/Cytoperm Kit (Becton Dickinson, Franklin Lakes, NJ). Then, the cells were stained with the antibodies as detailed in Table S1. Stained undifferentiated hiPSCs and stage 4 day 0 cells were used as negative controls for gating.

Implantation Experiments

All animal experiments were performed in accordance with the Guidelines for Animal Experiments of Kyoto University. Male 7- to 13-week-old NOD.CB17-Prkdcscid/J mice were anesthetized with inhalable isoflurane and received implants of hiPSC-derived cell aggregates after stage 4 under a kidney subcapsule. After the cells on stage 4 day 4 formed aggregates (3 × 10^{4} cells/aggregate), they were cultured in stage 4 medium with or without an ALK5 inhibitor for an additional 2 days before implantation. All metabolic analyses were performed in conscious and restrained animals.

(C and D) Cell density (C) and mRNA expression of *NKX6.1* (D) in cells after 4 days of pancreatic endoderm induction.
(E) PDX1^{+} posterior foregut cells were re-seeded at various cell densities (4–48 × 10^{4} cells/cm^{2}). The next day, cells were cultured in stage 4 medium with or without Y-27632 (50 μM) or Blebbistatin (5 μM). After 4-day culture, the relationship between cell density and the proportion of PDX1^{+}NKX6.1^{+} cells was analyzed by flow cytometry. The scatterplot indicates data from three independent experiments.
(F) A model showing the regulation of pancreatic endoderm cell differentiation. Note that the involvement of the ROCK-NM II pathway in the maintenance of cellular morphology was not analyzed in this study.
Data are presented as the mean ± SD from three independent experiments in (A), (C), and (D). *$p < 0.05$, **$p < 0.01$, ***$p < 0.001$, versus water or DMSO. $^{\dagger}p < 0.05$, versus mitomycin C. n/a, not applicable.

SUPPLEMENTAL INFORMATION

Supplemental Information includes Supplemental Experimental Procedures, four figures, and two tables and can be found with this article online at http://dx.doi.org/10.1016/j.stemcr.2017.07.005.

AUTHOR CONTRIBUTIONS

T.T. wrote the first draft of the manuscript. T.T., A.K., and K.O. contributed to the manuscript edits and revisions. T.T. designed, directed, and interpreted the experiments. T.T., A.K., H.T., T.A., A.M., Y.H., M.N., and A.W. performed the experiments. K.O. supervised the study.

ACKNOWLEDGMENTS

This work was supported in part by funding from the Takeda Science Foundation to T.T., the Suzuken Memorial Foundation to T.T., the Mitsui Life Social Welfare Foundation to K.O., the Life Science Foundation of Japan to K.O., the Japan Society for the Promotion of Science (JSPS) through its “Funding Program for World-Leading Innovative R&D on Science and Technology (FIRST Program)” to K.O. and Scientific Research (C) (JSPS KAKENHI grant number 15K09385) to T.T., and the Japan Agency for Medical Research and Development (AMED) through its research grant “Core Center for iPS Cell Research, Research Center Network for Realization of Regenerative Medicine” to K.O. The authors thank Dr. Kanae Mitsunaga for her technical assistance in the flow cytometry analysis, Dr. Yasunori Sato for assistance with statistical analyses, and Dr. Peter Karagiannis for reading the manuscript.

Received: August 14, 2016
Revised: July 4, 2017
Accepted: July 5, 2017
Published: August 8, 2017

REFERENCES

Amano, M., Ito, M., Kimura, K., Fukata, Y., Chihara, K., Nakano, T., Matsuura, Y., and Kaibuchi, K. (1996). Phosphorylation and activation of myosin by Rho-associated kinase (Rho-kinase). J. Biol. Chem. *271*, 20246–20249.

Connelly, J.T., Gautrot, J.E., Trappmann, B., Tan, D.W., Donati, G., Huck, W.T., and Watt, F.M. (2010). Actin and serum response factor transduce physical cues from the microenvironment to regulate epidermal stem cell fate decisions. Nat. Cell Biol. *12*, 711–718.

Cortijo, C., Gouzi, M., Tissir, F., and Grapin-Botton, A. (2012). Planar cell polarity controls pancreatic beta cell differentiation and glucose homeostasis. Cell Rep. *2*, 1593–1606.

Doe, C., Bentley, R., Behm, D.J., Lafferty, R., Stavenger, R., Jung, D., Bamford, M., Panchal, T., Grygielko, E., Wright, L.L., et al. (2007). Novel Rho kinase inhibitors with anti-inflammatory and vasodilatory activities. J. Pharmacol. Exp. Ther. *320*, 89–98.

Dupont, S., Morsut, L., Aragona, M., Enzo, E., Giulitti, S., Cordenonsi, M., Zanconato, F., Le Digabel, J., Forcato, M., Bicciato, S., et al. (2011). Role of YAP/TAZ in mechanotransduction. Nature *474*, 179–183.

Fujita, T., Kitaura, F., and Fujii, H. (2015). A critical role of the Thy28-MYH9 axis in B cell-specific expression of the Pax5 gene in chicken B cells. PLoS One *10*, e0116579.

Jacobs, M., Hayakawa, K., Swenson, L., Bellon, S., Fleming, M., Taslimi, P., and Doran, J. (2006). The structure of dimeric ROCK I reveals the mechanism for ligand selectivity. J. Biol. Chem. *281*, 260–268.

Kelly, O.G., Chan, M.Y., Martinson, L.A., Kadoya, K., Ostertag, T.M., Ross, K.G., Richardson, M., Carpenter, M.K., D’Amour, K.A., Kroon, E., et al. (2011). Cell-surface markers for the isolation of pancreatic cell types derived from human embryonic stem cells. Nat. Biotechnol. *29*, 750–756.

Kesavan, G., Sand, F.W., Greiner, T.U., Johansson, J.K., Kobberup, S., Wu, X., Brakebusch, C., and Semb, H. (2009). Cdc42-mediated tubulogenesis controls cell specification. Cell *139*, 791–801.

Maharam, E., Yaport, M., Villanueva, N.L., Akinyibi, T., Laudier, D., He, Z., Leong, D.J., and Sun, H.B. (2015). Rho/Rock signal transduction pathway is required for MSC tenogenic differentiation. Bone Res. *3*, 15015.

Nobusue, H., Onishi, N., Shimizu, T., Sugihara, E., Oki, Y., Sumikawa, Y., Chiyoda, T., Akashi, K., Saya, H., and Kano, K. (2014). Regulation of MKL1 via actin cytoskeleton dynamics drives adipocyte differentiation. Nat. Commun. *5*, 3368.

Nostro, M.C., Sarangi, F., Yang, C., Holland, A., Elefanty, A.G., Stanley, E.G., Greiner, D.L., and Keller, G. (2015). Efficient generation of NKX6-1+ pancreatic progenitors from multiple human pluripotent stem cell lines. Stem Cell Reports *4*, 591–604.

Ogawa, S., Surapisitchat, J., Virtanen, C., Ogawa, M., Niapour, M., Sugamori, K.S., Wang, S., Tamblyn, L., Guillemette, C., Hoffmann, E., et al. (2013). Three-dimensional culture and cAMP signaling promote the maturation of human pluripotent stem cell-derived hepatocytes. Development *140*, 3285–3296.

Pagliuca, F.W., Millman, J.R., Gurtler, M., Segel, M., Van Dervort, A., Ryu, J.H., Peterson, Q.P., Greiner, D., and Melton, D.A. (2014). Generation of functional human pancreatic beta cells in vitro. Cell *159*, 428–439.

Petzold, K.M., Naumann, H., and Spagnoli, F.M. (2013). Rho signalling restriction by the RhoGAP Stard13 integrates growth and morphogenesis in the pancreas. Development *140*, 126–135.

Rezania, A., Bruin, J.E., Xu, J., Narayan, K., Fox, J.K., O’Neil, J.J., and Kieffer, T.J. (2013). Enrichment of human embryonic stem cell-derived NKX6.1-expressing pancreatic progenitor cells accelerates the maturation of insulin-secreting cells in vivo. Stem Cells *31*, 2432–2442.

Rezania, A., Bruin, J.E., Arora, P., Rubin, A., Batushansky, I., Asadi, A., O’Dwyer, S., Quiskamp, N., Mojibian, M., Albrecht, T., et al. (2014). Reversal of diabetes with insulin-producing cells derived in vitro from human pluripotent stem cells. Nat. Biotechnol. *32*, 1121–1133.

Russ, H.A., Parent, A.V., Ringler, J.J., Hennings, T.G., Nair, G.G., Shveygert, M., Guo, T., Puri, S., Haataja, L., Cirulli, V., et al. (2015). Controlled induction of human pancreatic progenitors produces functional beta-like cells in vitro. EMBO J. *34*, 1759–1772.

Schyschka, L., Sanchez, J.J., Wang, Z., Burkhardt, B., Muller-Vieira, U., Zeilinger, K., Bachmann, A., Nadalin, S., Damm, G., and Nussler, A.K. (2013). Hepatic 3D cultures but not 2D cultures preserve specific transporter activity for acetaminophen-induced hepatotoxicity. Arch. Toxicol. *87*, 1581–1593.

Shih, H.P., Panlasigui, D., Cirulli, V., and Sander, M. (2016). ECM signaling regulates collective cellular dynamics to control pancreas branching morphogenesis. Cell Rep. *14*, 169–179.

Sun, Y., Yong, K.M., Villa-Diaz, L.G., Zhang, X., Chen, W., Philson, R., Weng, S., Xu, H., Krebsbach, P.H., and Fu, J. (2014). Hippo/YAP-mediated rigidity-dependent motor neuron differentiation of human pluripotent stem cells. Nat. Mater. *13*, 599–604.

Toyoda, T., Mae, S., Tanaka, H., Kondo, Y., Funato, M., Hosokawa, Y., Sudo, T., Kawaguchi, Y., and Osafune, K. (2015). Cell aggregation optimizes the differentiation of human ESCs and iPSCs into pancreatic bud-like progenitor cells. Stem Cell Res. *14*, 185–197.

Villasenor, A., Chong, D.C., Henkemeyer, M., and Cleaver, O. (2010). Epithelial dynamics of pancreatic branching morphogenesis. Development *137*, 4295–4305.

Wang, H., Rusielewicz, T., Tewari, A., Leitman, E.M., Einheber, S., and Melendez-Vasquez, C.V. (2012). Myosin II is a negative regulator of oligodendrocyte morphological differentiation. J. Neurosci. Res. *90*, 1547–1556.

Stem Cell Reports, Volume *9*

Supplemental Information

Rho-Associated Kinases and Non-muscle Myosin IIs Inhibit the Differentiation of Human iPSCs to Pancreatic Endoderm

Taro Toyoda, Azuma Kimura, Hiromi Tanaka, Tomonaga Ameku, Atsushi Mima, Yurie Hirose, Masahiro Nakamura, Akira Watanabe, and Kenji Osafune

Figure S1

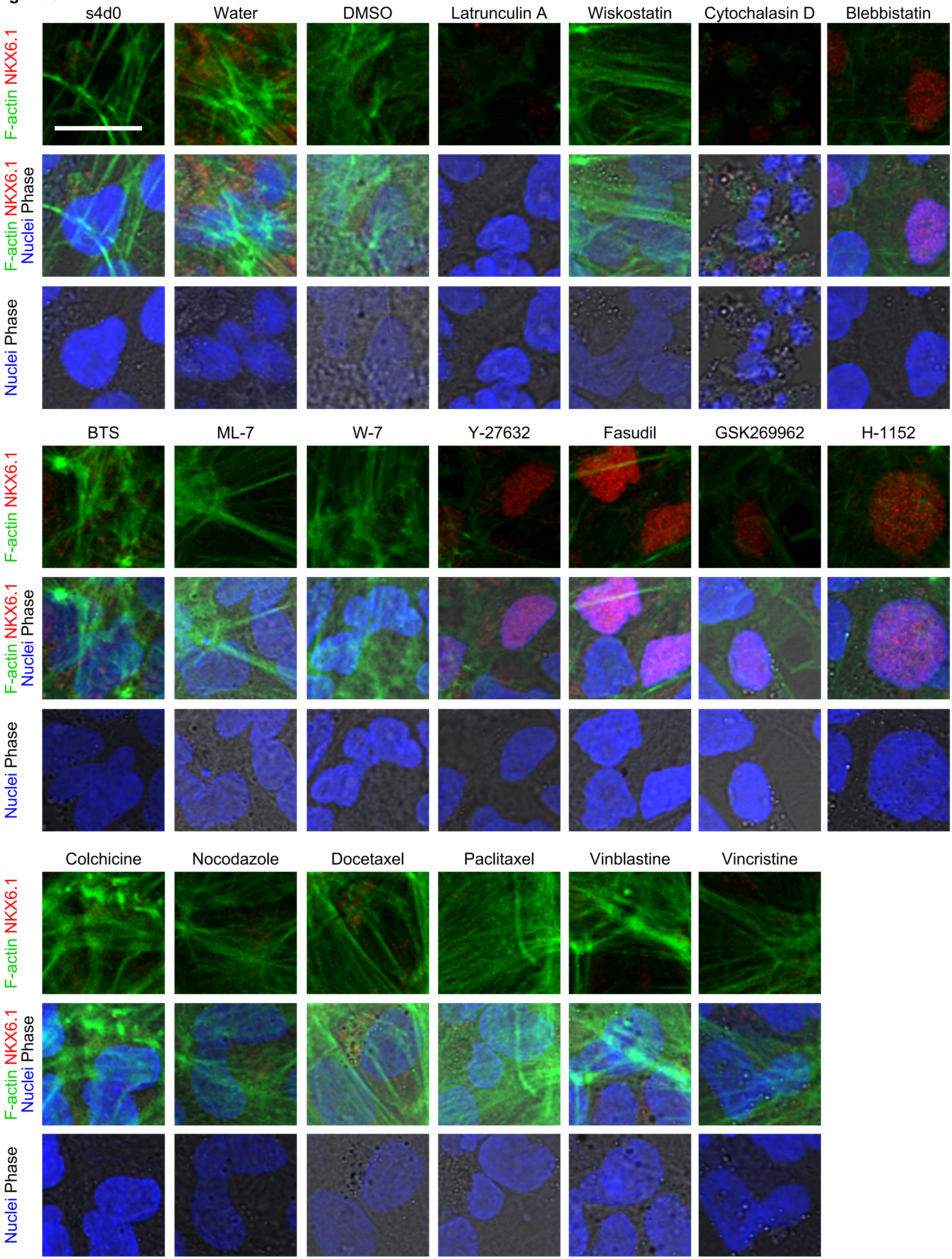

s4d0
Water
DMSO
Latrunculin A
Wiskostatin
Cytochalasin D
Blebbistatin
F-actin NKX6.1
F-actin Nuclei NKX6.1 Phase
Nuclei Phase
BTS
ML-7
W-7
Y-27632
Fasudil
GSK269962
H-1152
Colchicine
Nocodazole
Docetaxel
Paclitaxel
Vinblastine
Vincristine

Figure S2

A

Name	Main targets	Other targets
Latrunculin A	Monomeric actin	
Wiskostatin	N-WASP	
Cytochalasin D	Filamentous actin	
Blebbistatin	Myosin II	
BTS	Skeletal muscle myosin II	
ML-7	MLCK	
W-7	Calmodulin, PDE1 and MLCK	
Y-27632	ROCK1	PKC, PKA, MLCK and PRK2
Fasudil	PKA and ROCK	
GSK269962	ROCK1 and ROCK2	
H-1152	ROCK2	CAMKII, PKG, Aurora A, PKA, PKC and MLCK
Colchicine	Tubulin	
Nocodazole	Tubulin	
Docetaxel	Microtubule	
Paclitaxel	Microtubule	
Vinblastine	Tubulin	
Vincristine	Tubulin	

B

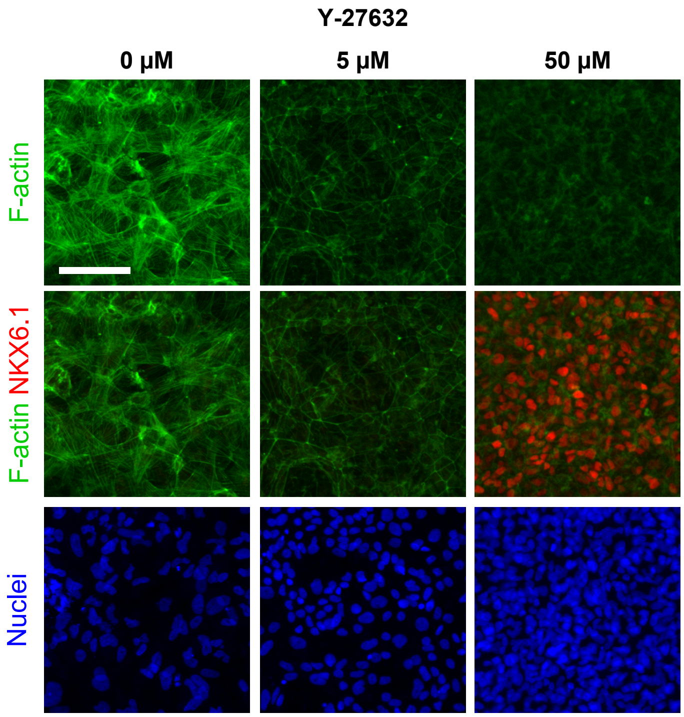

C

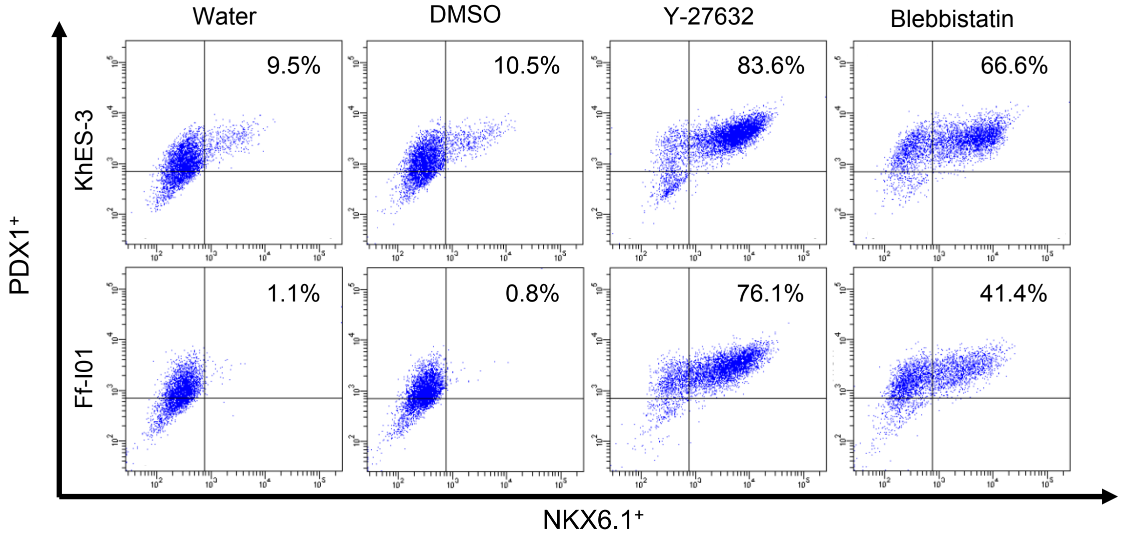

Figure S3

A

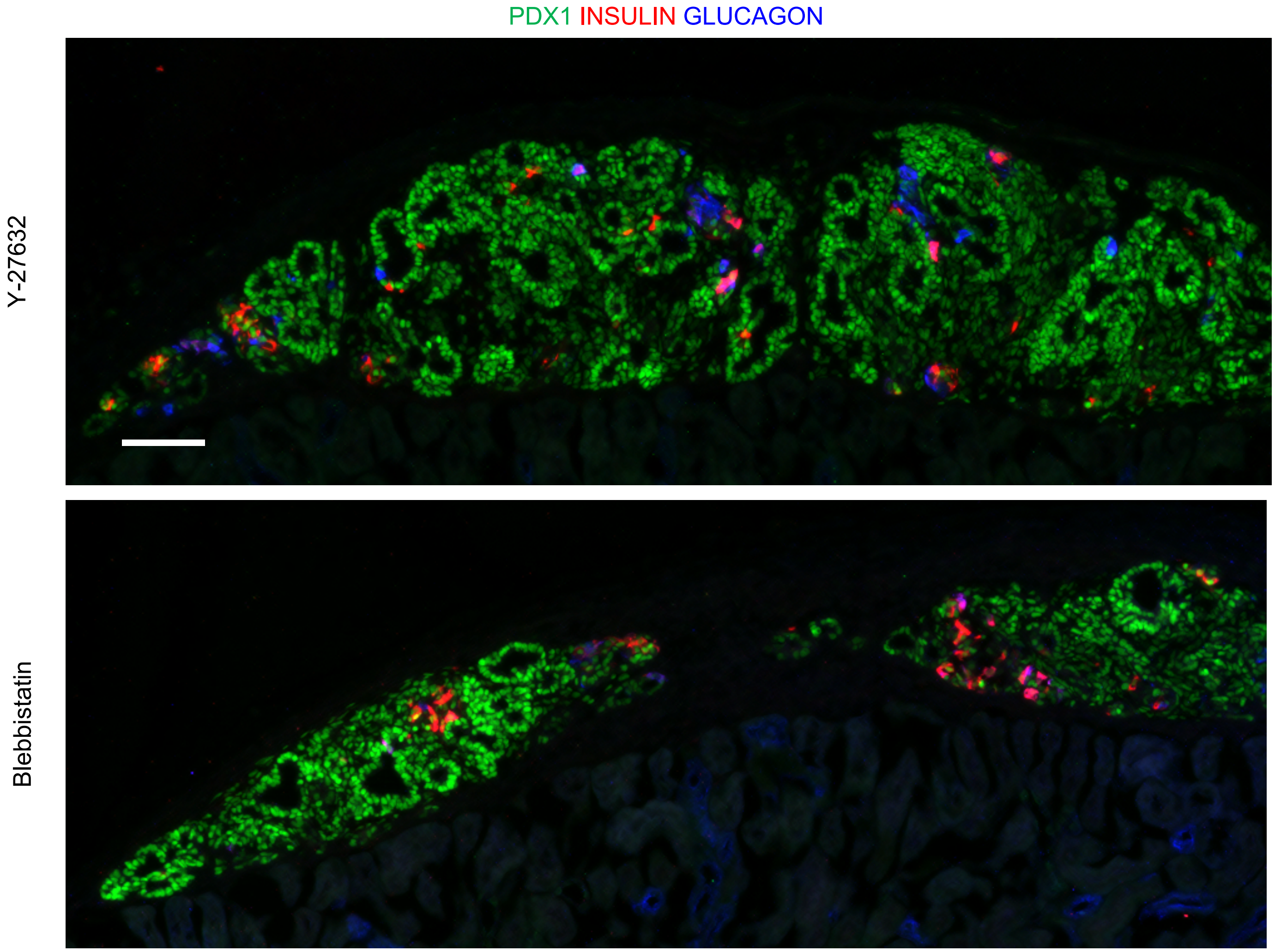

B

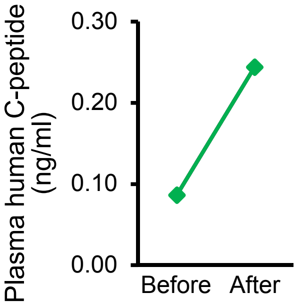

C

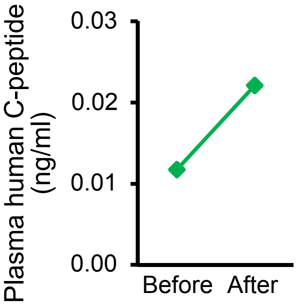

D

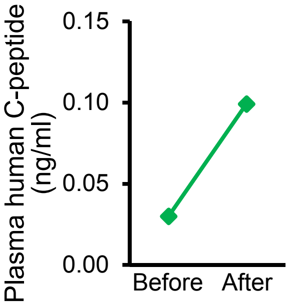

Figure S4

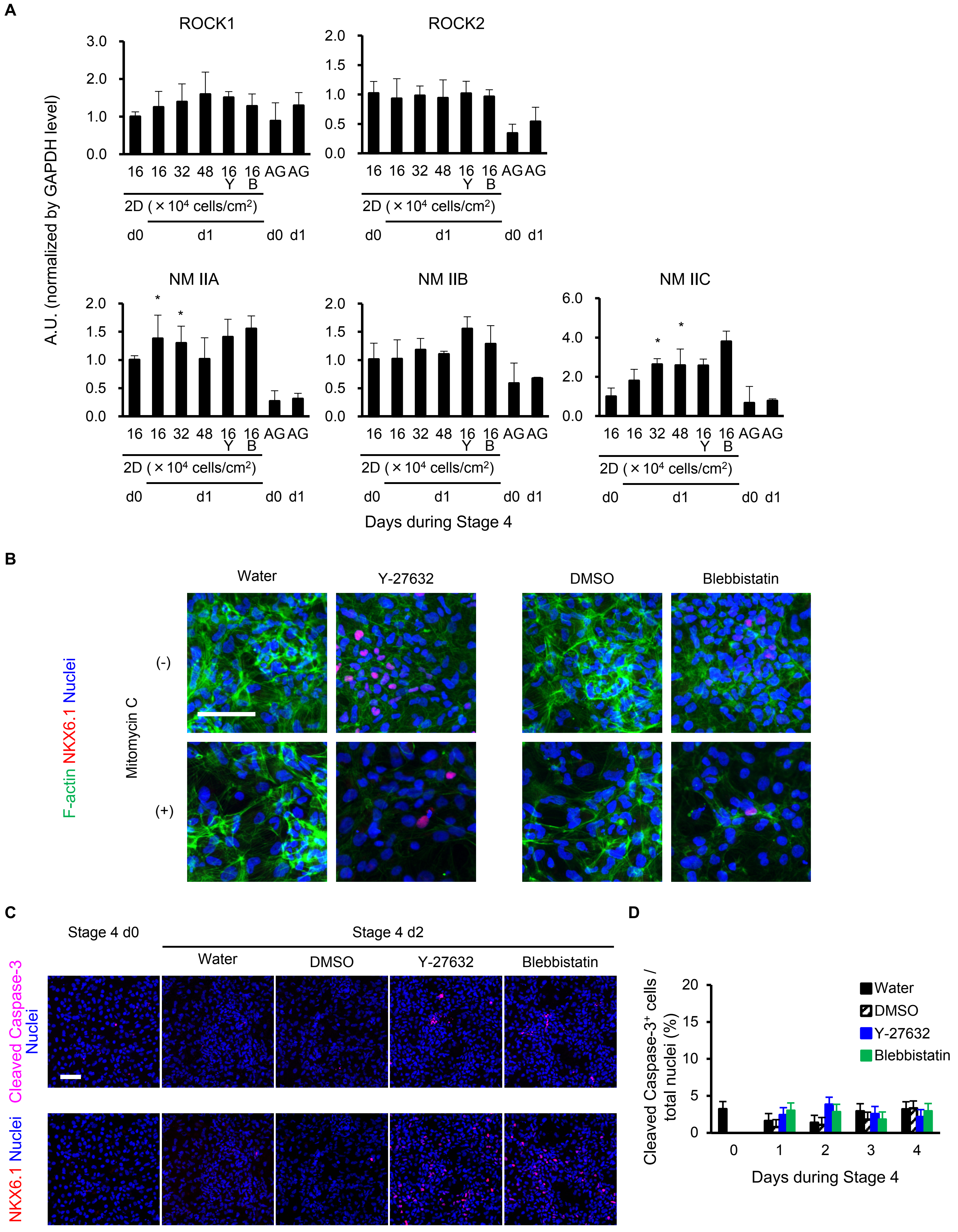
A
ROCK1
ROCK2
NM IIA
NM IIB
NM IIC
A.U. (normalized by GAPDH level)
3.0
2.0
1.5
1.0
0.5
0.0
6.0
4.0
16 16 32 48 16 16 AG AG
Y B
2D (×10⁴ cells/cm²)
d0 d1 d0 d1
Days during Stage 4
B
Water
Y-27632
DMSO
Blebbistatin
F-actin NKX6.1 Nuclei
Mitomycin C
(-)
(+)
C
Stage 4 d0
Stage 4 d2
Water
DMSO
Y-27632
Blebbistatin
Cleaved Caspase-3 Nuclei
NKX6.1 Nuclei
D
Cleaved Caspase-3⁺ cells / total nuclei (%)
20
15
10
5
0
0 1 2 3 4
Days during Stage 4
Water
DMSO
Y-27632
Blebbistatin

Supplemental Figure Legends

Figure S1 NKX6.1$^+$ cell induction does not always accompany disruption of actin fibers or cell shape changes. Related to Figure 1.

hiPSC-derived PDX1$^+$ posterior foregut cells were cultured with KGF (100 ng/ml), NOGGIN (100 ng/ml), EGF (50 ng/ml) and several cytoskeletal modulators: Latrunculin A (10 nM), Wiskostatin (1 μM), Cytochalasin D (50 μM), Blebbistatin (5 μM), BTS (50 μM), ML-7 (5 μM), W-7 (5 μM), Y-27632 (50 μM), Fasudil (50 μM), GSK269962 (10 μM), H-1152 (10 μM), Colchicine (10 nM), Nocodazole (100 nM), Docetaxel (10 μM), Paclitaxel (5 μM), Vinblastine (10 nM) and Vincristine (10 nM). The cells were fixed on Stage 4 day 4 and stained with F-actin (green), NKX6.1 (red) and nuclei (blue). Cell shapes were observed by phase contrast images. Representative images of three independent experiments. Note that NKX6.1$^+$ cells were not induced by any concentration of Latrunculin A (0.005, 0.01, 0.05, 0.1, 0.5, 1, 5 and 10 μM), Wiskostatin (0.01, 0.05, 0.1, 0.5, 1, 5, 10, 20 and 50 μM), Cytochalasin D (0.005, 0.01, 0.05, 0.1, 0.5, 1, 5, 10, 20 and 50 μM), BTS (0.01, 0.05, 0.1, 0.5, 1, 5, 10, 20, 30, 50, 80 and 100 μM), ML-7 (0.01, 0.05, 0.1, 0.5, 1, 5, 10, 20, 30 and 50 μM), W-7 (0.01, 0.05, 0.1, 0.5, 1, 5, 10, 20, 50, 80 and 100 μM), Colchicine (0.01, 0.05, 0.1, 0.5, 1, 5, 10, 20, 50, 80 and 100 μM), Nocodazole (0.01, 0.05, 0.1, 0.5, 1, 5, 10, 20 30 and 50 μM), Docetaxel (0.01, 0.05, 0.1, 0.5, 1, 5, 10, 20, 50 and 100 μM), Paclitaxel (0.01, 0.05, 0.1, 0.5, 1, 5, 10, 20, 50, 100 μM), Vinblastine (0.01, 0.05, 0.1, 0.5, 1, 5, 10, 20, 50, 80 and 100 μM) or Vincristine (0.01, 0.05, 0.1, 0.5, 1, 5, 10, 20, 50, 80 and 100 μM) (data not shown). Scale bar, 20 μm. F-actin, filamentous actin.

Figure S2 NKX6.1$^+$ cell induction does not always accompany disruption of actin fibers. Related to Figures 1 and 2.

(A) A list of main and other targets of the cytoskeletal modulators used in Figure 1. The targets are based on the Tocris Bioscience website (Bristol, United Kingdom, https://www.tocris.com/). (B) Representative images of dose dependent NKX6.1$^+$ cell induction in Y-27632-treated cells. hiPSC-derived PDX1$^+$ posterior foregut cells were cultured with KGF (100 ng/ml), NOGGIN (100 ng/ml), EGF (50 ng/ml) and Y-27632 (0, 5 and 50 μM). The cells were fixed on Stage 4 day 4 and stained with F-actin (green), NKX6.1 (red) and nuclei (blue). Low dose of Y-27632 treatment (5 μM) was effective at disrupting actin fibers but failed to induce NKX6.1$^+$ cells. Scale bar, 100 μm. (C) ROCK-NM II inhibitors effectively induced PDX1$^+$NKX6.1$^+$ cells from multiple hESC/iPSC lines. The proportion of PDX1$^+$NKX6.1$^+$ cells was quantified by flow cytometry after 4-day induction of pancreatic endoderm cells in the presence or absence of Y-27632 (50 μM) or Blebbistatin (5 μM) at Stage 4 in hESC line KhES-3 and hiPSC line Ff-I01. Abbreviations for the list in (A) are as follows. CAMKII, Ca^{2+}-calmodulin-dependent protein kinase II; MLCK, myosin light-chain kinase; N-WASP, neural Wiskott-Aldrich syndrome protein; PDE1, Ca^{2+}-calmodulin-dependent phosphodiesterase; PKA, cyclic AMP-dependent protein kinase; PKC, protein kinase C; PKG, cyclic GMP-dependent protein kinase; PRK2, Protein kinase C-related kinase 2; ROCK1, Rho-associated protein kinase 1; ROCK2, Rho-associated protein kinase 2.

Figure S3 PDX1$^+$NKX6.1$^+$ cells generated with Y-27632 or Blebbistatin develop into pancreatic epithelia *in vivo*. Related to Figure 2.

(A) Representative cryosection images of grafts 30 days after implantation were stained for the indicated markers from three individual mice for each group in one independent cohort of implantation experiments. Pancreatic endoderm cells induced with or without Y-27632 or Blebbistatin treatment on Stage 4 day 4 were dissociated and re-seeded to form cellular aggregates (3×10^4 cells/aggregate). A total of one million cells on Stage 4 day 6 were implanted under the kidney subcapsule of NOD-SCID mice. PDX1$^+$NKX6.1$^+$ cells induced with Y-27632 or Blebbistatin developed into branched pancreatic epithelia *in vivo*. Note that we could not observe the engraftment of control cells cultured at low density (1.6×10^5 cells/cm^2) without Y-27632 or Blebbistatin for four days at Stage 4 (data not shown). Scale bar, 100 μm. (B, C and D) Pancreatic endoderm cells induced with Blebbistatin treatment on Stage 4 day 4 were dissociated and re-seeded to form cellular aggregates (3×10^4 cells/aggregate). A total of three to four million cells on Stage 4 day 6 that had been pre-treated with an ALK5 inhibitor for 2 days were implanted under the kidney subcapsule of NOD-SCID mice. The plasma human C-peptide levels after 16 hours of fasting (Before) and 30 min after subsequent glucose injection (3.0 g/kg body weight, i.p.) (After) in the host mice were examined on days 120 (B), 151 (C) and 133 (D) after implantation. Data in (B)-(D) are from an individual mouse from two independent cohorts of implantation experiments.

Figure S4 Y-27632 and Blebbistatin induce NKX6.1 expression without increasing cellular proliferation or suppressing apoptosis. Related to Figures 3 and 4.

(A) Immunoblot quantification of ROCK1, ROCK2, NM IIA, NM IIB and NM IIC in Stage 4 cells. hiPSC-derived

PDX1^{+} posterior foregut cells on Stage 3 day 2 were dissociated and re-seeded either for monolayer cultures (16–48×10^{4} cells/cm^{2}, 2D) or to form cellular aggregates (3×10^{4} cells/aggregate, AG). The cells were continuously cultured with Stage 3 treatment, except for the addition of 10 μM Y-27632. On the next day, the cells were cultured with Stage 4 with or without ROCK-NM II inhibitors. Protein abundance is normalized to the average protein abundance of re-seeded cells (16×10^{4} cells/cm^{2}) on Stage 4 day 0. (B) Representative images of Stage 4 day 4 cells pre-treated with or without mitomycin C (47 μM, 2 h). On Stage 4 day 4, NKX6.1^{+} cells were observed in Y-27632 (50 μM)- or Blebbistatin (5 μM)-treated cells after mitomycin C treatment, but not in their corresponding controls. (C and D) Apoptotic cells were not reduced by Y-27632 or Blebbistatin treatment. (C) Representative images of cells immunostained with an apoptotic marker, cleaved Caspase-3, from three independent experiments. (D) The percentage of cleaved Caspase-3^{+} cells in (C) was analyzed by manual counting. Data are presented as the mean ± S.D. from three independent experiments (n=3) in (A) and (D). *, $P < 0.05$ versus AG on day 1. Scale bar, 100 μm. Y, Y-27632; B, Blebbistatin.

Supplemental Tables

Table S1. Antibodies used in this study.

**Antigen**	**Species**	**Source**	**Dilution**
PDX1	Goat	*AF2419, R&D Systems	1:200
NKX6.1	Mouse	*F55A12, University of Iowa	1:100
Insulin	Guinea pig	A0564, Dako	1:200
Glucagon	Mouse	G2654, Sigma-Aldrich	1:200
Nucleus	N/A	Hoechst 33342; H3570, Invitrogen	1:200
ROCK1	Rabbit	4035, Cell Signaling Technology	1:1000
ROCK2	Mouse	610624, BD Transduction Laboratories	1:2000
NM IIA	Rabbit	M8064, Sigma	1:4000
NM IIB	Rabbit	M7939, Sigma	1:4000
NM IIC	Rabbit	8189, Cell Signaling Technology	1:1000
GAPDH	Mouse	014-25524, Wako	1:5000
Cleaved Caspase-3	Rabbit	9661, Cell Signaling Technology	1:200
pMLC2 Ser^{19}	Rabbit	3671, Cell Signaling Technology	1:200

*: antibodies used for flow cytometry

Table S2. Gene names and primer sequences for qRT-PCR

**Gene name**	**Gene Symbol**	**Forward primer**	**Reverse primer**
Pancreas specific transcription factor, 1a	*PTF1A*	CCCCAGCGACCCTGATTA	GGACACAAACTCAAATGGTGG
NK6 homeobox 1	*NKX6.1*	ATTCGTTGGGGATGACAGAG	TGGGATCCAGAGGCTTATTG
Glyceraldehyde-3-phosphate dehydrogenase	*GAPDH*	GAAGGTGAAGGTCGGAGTC	GAAGATGGTGATGGGATTTC
Rho associated coiled-coil containing protein kinase 1	*ROCK1*	CTGCAACTGGAACTCAACCA	TTCTACCAATTGCGCTTGC
Rho-associated coiled-coil containing protein kinase 2	*ROCK2*	CCTGTCAAGCGTGGTAATGA	CGAATCTGGCTCTCTTCAGC
myosin, heavy chain 9, non-muscle	*MYH9*	GGGCACTGTCAAGTCCAAGT	AGCAGCACATCCTTCAGCTT
myosin, heavy chain 10, non-muscle	*MYH10*	GCAGAACAAGGAGCTGAAGG	TGCGACGGACTAATTTGTTG
myosin, heavy chain 14, non-muscle	*MYH14*	CCACAAGATGACCATTGCTG	CAGCTTTCCAGAGAGGATGC

Supplemental Experimental Procedures

hESC/iPSC culture and differentiation

The maintenance culture of a human embryonic stem cell (hESC) line, KhES-3 (Suemori et al., 2006), and a human induced pluripotent stem cell (hiPSC) line, 585A1 (Kajiwara et al., 2012), was performed as described previously (Toyoda et al., 2015). The peripheral blood-derived iPSC line Ff-I01 was generated at the Center for iPS Cell Research and Application (CiRA), Kyoto University. For on-feeder cultures, cells were grown on feeder layers of mitomycin C-treated SNL 76/7 (ECACC 07032801) in media containing Primate ES medium (ReproCELL, Yokohama, Japan) supplemented with 500 U/ml penicillin/streptomycin (P/S, Thermo Fisher Scientific, Waltham, MA) and 4 ng/ml recombinant human basic fibroblast growth factor (bFGF; Wako, Osaka, Japan). For routine passaging, hESC/iPSC colonies were dissociated by an enzymatic method with CTK dissociation solution consisting of 0.25% trypsin (Thermo Fisher Scientific), 0.1% collagenase IV (Thermo Fisher Scientific), 20% knockout serum replacement (KSR; Thermo Fisher Scientific) and 1 mM $CaCl_2$ in phosphate buffered saline (PBS) and split at a ratio between 1:6 and 1:8. For feeder-free cultures, cells were maintained with Essential 8 medium (Thermo Fisher Scientific) according to the manufacturer's instructions. For Ff-I01, cells were maintained with StemFit AK03 (Ajinomoto, Tokyo, Japan) on iMatrix-511 (Nippi, Tokyo, Japan) according to the manufacturer's instructions. Experiments with hESCs/iPSCs were approved by the ethics committee of the Department of Medicine and Graduate School of Medicine, Kyoto University. Cells were directed into key stages of pancreatic development, including definitive endoderm (Stage 1), primitive gut tube (Stage 2), posterior foregut (Stage 3) and pancreatic endoderm (Stage 4). The final protocol follows Figure 1A.

Stage 1: hESC/iPSC colonies grown on a SNL-feeder layer were first deprived of feeder cells (Suemori et al., 2006). Then, the cells were dissociated into single cells as described previously (Toyoda et al., 2015). The cells were resuspended with Stage 1 medium containing RPMI 1640 medium (NACALAI TESQUE, Kyoto, Japan) supplemented with 2% (vol/vol) B-27 Serum-Free Supplement (B27, Thermo Fisher Scientific), 50 U/ml P/S, 100 ng/ml recombinant human/mouse/rat activin A (R&D Systems, Minneapolis, MN), 3 μM CHIR99021 (Axon Medchem, Groningen, Netherlands) and 10 μM Y-27632 (Wako), seeded on BD Matrigel Basement Membrane Matrix Growth Factor Reduced (Matrigel, Becton Dickinson, Franklin Lakes, NJ)-coated plates at a density of 1×10^5 cells/cm^2 and cultured for one day. For the next two days, the cells were cultured in RPMI 1640 medium with 2% B27, 50 U/ml P/S, 100 ng/ml activin A and 1 μM CHIR99021. The day after that, the cells were cultured in RPMI 1640 medium with 2% B27, 50 U/ml P/S and 100 ng/ml activin A.

Stage 2: The cells were exposed to Improved MEM Zinc Option (iMEM) medium (Thermo Fisher Scientific) supplemented with 1% B27, 100 U/ml P/S (iMEM-B27) and 50 ng/ml keratinocyte growth factor (KGF; R&D Systems) for four days.

Stage 3: The cultures were continued for two days in iMEM-B27 with 0.5 μM 3-Keto-N-aminoethyl-N′-aminocaproyldihydrocinnamoyl cyclopamine (KAAD-CYC; Toronto Research Chemicals, Toronto, Canada), 0.5 nM 4-[(E)-2-(5,6,7,8-Tetrahydro-5,5,8,8-tetramethyl-2-naphthalenyl)-1-propenyl]-benzoic acid (TTNPB, Santa Cruz Biotechnology, Dallas, TX), 50 ng/ml KGF and 100 ng/ml NOGGIN (PeproTech, Rocky Hill, NJ). On the next day, the cells were dissociated into single cells by gentle pipetting after treatment with 0.25% trypsin–EDTA. Then, the cells were re-seeded on Matrigel-coated plates at a density of 1.6×10^5 cells/cm^2. The same inducing factors were used as described above, except for the addition of 10 μM Y-27632 to the Stage 3 treatment.

Stage 4: The cells were cultured for 1-8 days in iMEM-B27 with 100 ng/ml KGF, 100 ng/ml NOGGIN, 50 ng/ml epidermal growth factor (EGF, R&D Systems) and cytoskeletal modulators, such as 50 μM Y-27632 or 5 μM (*S*)-(-)-Blebbistatin (Blebbistatin, Toronto Research Chemicals). To evaluate various cytoskeletal modulators, the cells were subjected to four days of treatment with Latrunculin A (10 nM, Santa Cruz Biotechnology), Wiskostatin (1 μM, Santa Cruz Biotechnology), Cytochalasin D (50 μM, Santa Cruz Biotechnology), Blebbistatin (5 μM), BTS (50 μM, Santa Cruz Biotechnology), ML-7 (5 μM, Santa Cruz Biotechnology), W-7 (5 μM, Wako), Y-27632 (50 μM), Fasudil (50 μM, NACALAI TESQUE), GSK269962 (10 μM, Wako), H-1152 (10 μM, Wako), Colchicine (10 nM, Wako), Nocodazole (100 nM, Wako), Docetaxel (10 μM, Sigma), Paclitaxel (5 μM, Wako), Vinblastine (10 nM, Wako) and Vincristine (10 nM, LKT Laboratories, Paul, MN). For mitomycin C treatment, the cells were pre-treated with mitomycin C (47 μM, 2 h, Kyowa Hakko Kirin, Tokyo, Japan) before the start of Stage 4 culture.

Immunostaining

The cells were fixed with 4% paraformaldehyde (PFA) for 20 min at 4 °C. Then, immunostaining was performed as described previously (Toyoda et al., 2015). The primary antibodies used are detailed in Table S1. Nuclei were stained with Hoechst 33342 (Thermo Fisher Scientific). Filamentous-actin (F-actin) was stained with Acti-stain

phalloidin (Cytoskeletal, Denver CO). The implanted grafts were fixed with 4% PFA for 1-2 days at 4 °C. After washing with PBS, the samples were equilibrated in a 10–30% sucrose solution at room temperature for 1 h and then mounted and frozen. The frozen blocks were sectioned at 10–30 µm, and immunostaining was performed after removing the mounting medium. For quantification of the NKX6.1$^+$ cell ratios, immunostained cells were analyzed using an image analyzer CellInsight NXT (Thermo Fisher Scientific). For quantification of the cleaved Caspase-3$^+$ cell rate, immunostained cells were analyzed by manual counting.

Flow cytometry

The cells were dissociated into single cells with 0.25% trypsin–EDTA treatment, fixed, permeabilized and blocked with BD Cytofix/Cytoperm Kit (Becton Dickinson). Then, the cells were stained with the antibodies detailed in Table S1. Stained undifferentiated hiPSCs and Stage 4 day 0 cells were used as negative controls for gating.

Quantitative real-time reverse transcription polymerase chain reaction (qRT-PCR)

Total RNA was isolated from the cells with an RNeasy kit (Qiagen, Hilden, Germany), and cDNA was prepared with a ReverTra Ace qPCR RT Master Mix (TOYOBO, Osaka, Japan) and oligo (dT)20 primer according to the manufacturer's instructions. The qRT-PCR analysis was carried out with SYBR Premix Ex Taq II (Takara, Otsu, Japan). The expression of each gene was normalized to the level of *glyceraldehyde-3-phosphate dehydrogenase* (*GAPDH*) expression. The primer sequences used are shown in Table S2.

Animal studies and implantation experiments

All animal experiments were performed in accordance with the Guidelines for Animal Experiments of Kyoto University. Male 7- to 14-week-old NOD.CB17-Prkdc^{scid}/J mice (NOD–SCID, Charles River Laboratories Japan, Yokohama, Japan) were maintained on a 12-h light/dark cycle with ad libitum access to a standard irradiated diet. Mice were anesthetized with inhalable isoflurane and received implants of hiPSC-derived cell aggregates after Stage 4. The cells on Stage 4 day 4 were dissociated, and cell aggregates (3×10^4 cells/aggregate) were formed. The cell aggregates were cultured in Stage 4 medium with or without an ALK5 inhibitor (Santa Cruz Biotechnology) for an additional two days before implantation. Then, 60-200 cell aggregates per mouse were implanted under a kidney subcapsule. At 30 days after implantation, serial sections of the grafts were examined by immunostaining, as described above. The graft function was assessed by measuring human C-peptide levels in mouse plasma in response to glucose administration. The mice were fasted for >5 h, and then a 30% glucose solution was administered by intraperitoneal injection at a dose of 3.0 g/kg body weight. Blood samples were collected prior to and at 30 min after the glucose administration via a tail vein to heparinized capillaries. The plasma human C-peptide levels were analyzed by an ELISA (Mercodia, Uppsala, Sweden) according to the manufacturer's instructions. All metabolic analyses were performed in conscious and restrained animals.

Immunoblots

The cells were processed with a rubber scraper in RIPA Buffer (Wako) with Protease Inhibitor Cocktail Set III (Wako) and Phosphatase Inhibitor Cocktail Solution I (Wako), and then subjected to Bioruptor UCD-250HSA (Tosyodenki, Kanagawa, Japan) on ice. After centrifugation (15,000 × *g*) at 4 °C for 15 min, the supernatant was collected. Lysate protein concentrations were determined by the Bradford method (Bradford, 1976) using the dye reagent XL-Bradford (KY-1040, APRO SCIENCE, Naruto, Japan). Lysates (2-4 g of protein) were separated by SDS-PAGE before immunoblotting (Laemmli, 1970). Antibody-bound proteins were visualized with chemiluminescence detection reagents (GE Healthcare, Buckinghamshire, U.K.) and detected using the LAS-4000 mini (Fujifilm, Tokyo, Japan). Images were quantitated by densitometry (Multi Gauge Ver. 3.2, Fujifilm). Protein expressions were quantified relative to the loading control and normalized by GAPDH levels. Fold increases are expressed relative to the average of the lysates from monolayer culture (1.6×10^5 cells/cm^2) on Stage 4 day 0. The primary antibodies used in this study are detailed in Table S1.

Statistics

Data are expressed as the means ± standard deviation or standard error. Statistical analyses were performed using ratio paired t test, one-way analysis of variance (ANOVA), two-way ANOVA or two-way repeated measure ANOVA (Prism7, GraphPad Software, San Diego). When differences between means were detected by one- or two-way analysis of variances, Dunnett’s or Tukey’s multiple comparison test as appropriate was used for post hoc testing. Differences between groups were considered significant when $p < 0.05$.

Supplemental References

Bradford, M.M. (1976). A rapid and sensitive method for the quantitation of microgram quantities of protein utilizing the principle of protein-dye binding. Anal Biochem *72*, 248-254.

Kajiwara, M., Aoi, T., Okita, K., Takahashi, R., Inoue, H., Takayama, N., Endo, H., Eto, K., Toguchida, J., Uemoto, S., *et al.* (2012). Donor-dependent variations in hepatic differentiation from human-induced pluripotent stem cells. Proc Natl Acad Sci U S A *109*, 12538-12543.

Laemmli, U.K. (1970). Cleavage of structural proteins during the assembly of the head of bacteriophage T4. Nature *227*, 680-685.

Suemori, H., Yasuchika, K., Hasegawa, K., Fujioka, T., Tsuneyoshi, N., and Nakatsuji, N. (2006). Efficient establishment of human embryonic stem cell lines and long-term maintenance with stable karyotype by enzymatic bulk passage. Biochem Biophys Res Commun *345*, 926-932.
